# G-quadruplex DNA structure is a positive regulator of *MYC* transcription

**DOI:** 10.1073/pnas.2320240121

**Published:** 2024-02-05

**Authors:** Isabel Esain-Garcia, Angie Kirchner, Larry Melidis, Rafael de Cesaris Araujo Tavares, Somdutta Dhir, Angela Simeone, Zutao Yu, Sarah K. Madden, Regina Hermann, David Tannahill, Shankar Balasubramanian

**Affiliations:** ^a^Cancer Research UK Cambridge Institute, University of Cambridge, Cambridge CB2 0RE, United Kingdom; ^b^Yusuf Hamied Department of Chemistry, University of Cambridge, Cambridge CB2 1EW, United Kingdom; ^c^School of Clinical Medicine, University of Cambridge, Cambridge CB2 0SP, United Kingdom

**Keywords:** G-quadruplex, DNA, *MYC*, transcription, epigenetics

## Abstract

DNA structure can regulate genome function. Four-stranded DNA G-quadruplex (G4) structures have been implicated in transcriptional regulation; however, previous studies have not directly addressed the role of an individual G4 within its endogenous cellular context. Using CRISPR to genetically abrogate endogenous G4 structure folding, we directly interrogate the G4 found within the upstream regulatory region of the critical human *MYC* oncogene. G4 loss leads to suppression of *MYC* transcription from the P1 promoter that is mediated by the deposition of a de novo nucleosome alongside alterations in RNA polymerase recruitment. We also show that replacement of the endogenous *MYC* G4 with a different G4 structure from the *KRAS* oncogene restores G4 folding and *MYC* transcription. Moreover, we demonstrate that the *MYC* G4 structure itself, rather than its sequence, recruits transcription factors and histone modifiers. Overall, our work establishes that G4 structures are important features of transcriptional regulation that coordinate recruitment of key chromatin proteins and the transcriptional machinery through interactions with DNA secondary structure, rather than primary sequence.

## Significance

DNA G-quadruplexes (G4s) are four-stranded DNA structures enriched in regulatory regions of the human genome; however, their functional role in transcription remains an incompletely answered question. Using CRISPR genome editing, genomic approaches for chromatin profiling, and biophysical assays, we demonstrate that a G4 structure folds endogenously within the upstream promoter region of the critical *MYC* oncogene to positively regulate transcription. Key transcription factors and chromatin proteins bind to the *MYC* promoter via preferential interaction with a G4 DNA structure, rather than with the duplex primary sequence. Overall, this study demonstrates how G4 structures, rather than DNA sequence, alter the local chromatin landscape and nucleosome occupancy to positively promote transcription.

**Fig. 1.**
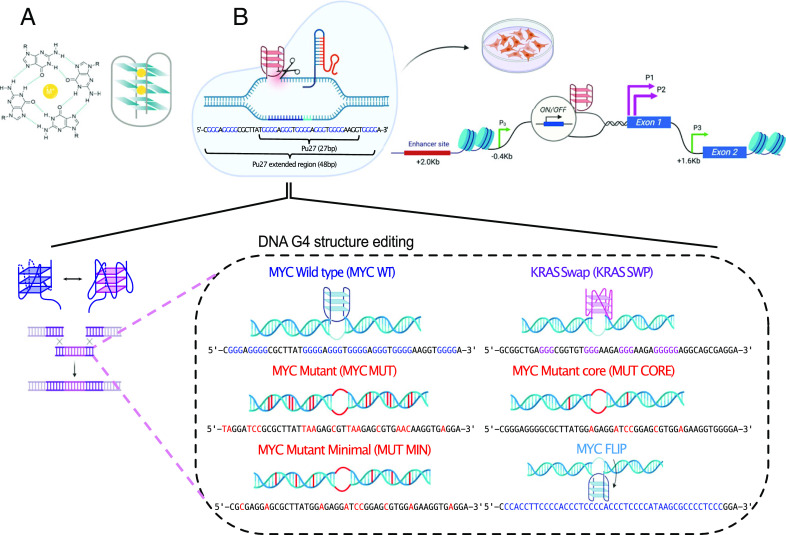
Genome editing of the *MYC* G4. (*A*) Illustration of a G-tetrad formed from guanines by Hoogsteen base-pairing coordinated by a central cation (*Left*). Stacking of G-tetrads to form a G4 structure (*Right*). (*B*) Experimental overview of CRISPR editing of the *MYC* G4, indicating the core 27-bp sequence (Pu27) consisting of five G-runs and the extended 48-bp sequence, consisting of eight G-runs in total (*Left*). Schematic of the *MYC* upstream regulatory region. The *MYC* G4 is ~100 bp upstream of promoter P1 in the nuclease hypersensitive site (NHE III_1_). The wild-type *MYC* G4 and edited sequences are shown. MYC MUT and MUT MIN cell lines contain destabilizing mutations in the eight G-runs to abolish G4 folding. MUT CORE contains point mutations in the core five G-runs within the 27 bp sequence. For KRAS SWP cells, the *MYC* G4 is replaced with the *KRAS* G4, similar in structure but dissimilar in sequence. In MYC FLIP cells, the G4 is switched to the opposite (template) strand.

DNA can adopt different secondary structures and influence genome function (1). A long-standing question has been whether and how DNA structure, rather than sequence, contributes to transcriptional mechanisms. One such structure of significant interest is the G-quadruplex (G4) formed from G-rich sequences that fold into stacked G-tetrads held together by Hoogsteen hydrogen bonding and stabilized by cations, especially potassium (Fig. 1*A*) (2). Sequence motifs encoding potential G4s are particularly enriched in human promoters, including many oncogenes (3–7). To date, G4 structure formation in cellular chromatin has been demonstrated using G4-structure-specific antibodies and small molecule probes (8–12). The mapping of G4 structures in chromatin has repeatedly shown a positive correlation of folded G4 structures in promoters with active transcription (13, 14). Consistent with this, biophysical and cell-based affinity enrichment experiments have shown that G4 structures themselves can bind to a range of transcription factors (TFs) and chromatin-binding proteins, including SP1 and CNBP (15–19). Furthermore, perturbation experiments in cells with G4-targeting small molecules have implicated G4s in transcriptional regulation (8, 20). In vitro experiments have suggested transcription can lead to G4 folding (21); however, G4s are still observed in cells when transcription is inhibited (22, 23). G4 formation appears to be related to chromatin context, which is coupled to the establishment of accessible chromatin and associated with specific histone modifications (13, 22).

The role of G4s in transcriptional regulation in an endogenous cellular context remains an unanswered question. Recent methodological innovations for detecting folded G4 DNA structures in situ, such as G4 CUT&Tag and Chem-map, together with genome editing now enable such experiments (9, 12, 24). Here, we focus on the *MYC* G4, located in the nuclease hypersensitive element (NHE III_1_) of the *MYC* oncogene, critical for cell cycle regulation, apoptosis, and cellular transformation (25). The *MYC* G4 is located immediately upstream (~100 bp) of the *MYC* P1 promoter, generally responsible for 15 to 20% of total *MYC* expression though can be greater in some cell types (26, 27) and is up-regulated in cancers such as Burkitt’s lymphoma (28–30).

The core 27-nucleotide sequence motif of the *MYC* G4 (Pu27) has been the subject of considerable biophysical and structural studies that demonstrate it folds in vitro into a very stable, parallel G4 structure under near-physiological conditions (31–33). Early work using reporter plasmids with *MYC* G4 sequences suggests a role for G4s in modulating transcription (34–36). However, such experiments used in vitro or plasmid assays and so did not directly assess transcription at the *MYC* locus in endogenous chromatin. Direct evidence for folded DNA G4 structures in cells has been lacking in previous experiments. Indeed, it is now evident that in cells, the majority of predicted G4 sequences do not actually result in detectable folded G4 structure (6, 13); therefore, detection of folded G4 structure at the locus of interest is essential to understand the relationship between structure and function. Experiments using small molecules that stabilize G4s have been deployed to manipulate *MYC* transcription (34, 37, 38), but these studies did not assess G4 structure formation or G4-target engagement by the small molecule(s) in cells, and so indirect effects on transcription cannot be ruled out. We set out to investigate the functional role of the *MYC* G4 structure in the endogenous cellular context and provide mechanistic insights into how this G4 affects the regulation of *MYC* transcription.

## Results

### Genome Editing Perturbs G-Quadruplex Formation at the *MYC* Promoter.

We used CRISPR (39) gene editing to perform structural perturbations at the endogenous *MYC* locus in human embryonic kidney (HEK293T) cells (Fig. 1*B*). The first perturbation was designed to abolish G4 structure formation by introducing point mutations in critical G bases. Mutation selection was guided by Cis-BP scoring (40), which informs on TF binding specificities and has been corroborated in cells by high-throughput ChIP-seq of chromatin-associated proteins (41). This ensured that key consensus TF motifs were not disrupted or introduced.

In designing destabilizing mutations, it was essential to consider Pu27 (27 bp, five G-runs) within the natural sequence context of an extended 48-bp region (i.e. MYC WT, eight G-runs), as it was previously shown that the flanking G-runs can contribute to G4 folding when central G-runs are mutated (31, 42, 43). Consistent with these observations, our results demonstrated that mutating Pu27 alone does not abrogate G4 formation based on in vitro biophysical measurements (*SI Appendix*, Figs. S1 and S2).

**Fig. 2.**
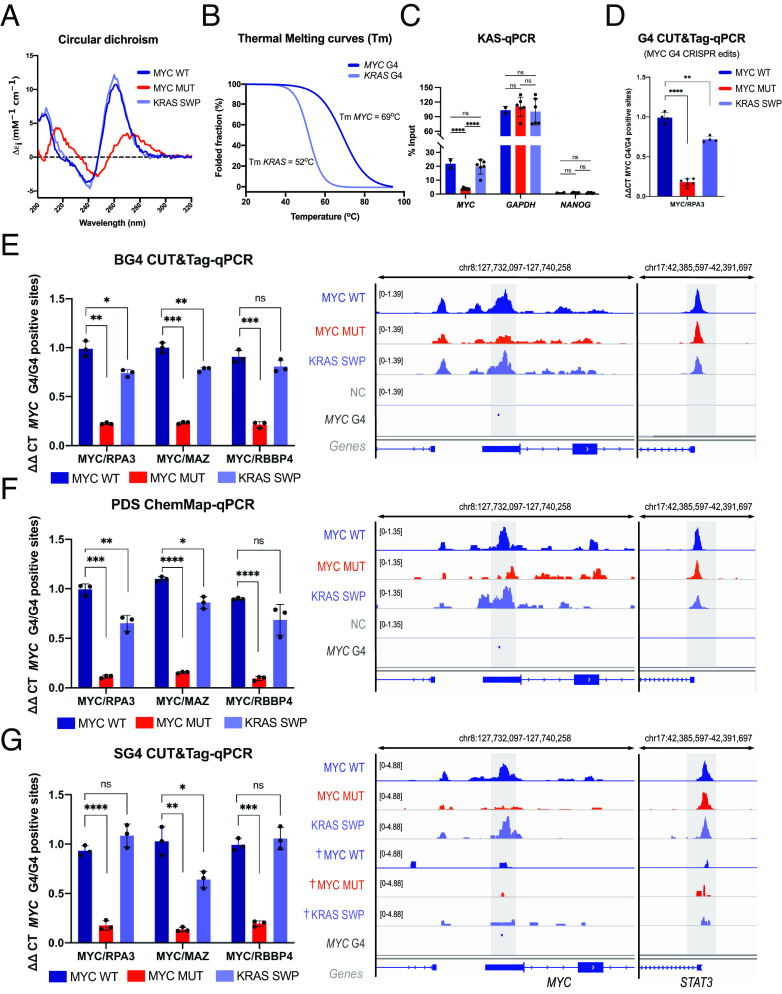
Structural perturbations of the *MYC* G4. (*A*) Circular dichroism (CD) spectra of MYC WT, MYC MUT, and KRAS SWP oligonucleotides (20 mM lithium cacodylate buffer, 10 mM KCl, pH 7.0). (*B*) CD melting curves for *MYC* and *KRAS* G4s, with T_m_ = 69 °C and T_m_ = 52 °C, respectively (20 mM lithium cacodylate buffer, 10 mM KCl, pH 7.0). (*C*) Kethoxal-assisted single-stranded-qPCR in MYC WT, MYC MUT, and KRAS SWP cells for the edited site, a positive G4-forming control (*GAPDH*) and G4-negative site (*NANOG*). Mean ± SD of MYC WT n = 1 and MYC MUT, KRAS SWP n = 3 independent biological samples measured twice as technical triplicates. (*D*) G4 CUT&Tag-qPCR with a G4 structure-specific antibody (BG4) for independently generated CRISPR clones for MYC WT, MYC MUT, and KRAS SWP cells. Mean ± SD of MYC MUT (N = 3), KRAS SWP (N = 2) independent clones, for n = 2 biological replicates, measured as technical triplicates. (*E*) BG4 CUT&Tag-qPCR (*Left*) for MYC WT, MYC MUT, and KRAS SWP cells relative to three G4s in control genes sites (*RPA3*, *MAZ*, *RBBP4*). G4 CUT&Tag sequencing profiles (Integrative Genomics Viewer, IGV tracks, *Right*) for the G4 edited site and the *STAT3* control site. Mean ± SD, n = 3 independent biological samples. (*F*) as in (*E*) but with PDS Chem-map RT-qPCR (*Left*) and sequencing (*Right*). (*G*) as in (*E*) but with a nanobody raised against the *MYC* G4 (SG4). CUT&Tag-qPCR (*Left*) calculated against the G4-positive sites of *RPA3*, *MAZ,* and *RBBP4*. Sequencing tracks (*Right*) showing the SG4 and SG4 mutant CUT&Tag signals. SG4 signal is observed in MYC WT and KRAS SWP, while no binding is observed to MYC MUT or with SG4 mutant nanobody (see ^†^MYC WT, ^†^MYC MUT, and ^†^KRAS SWP). *P*-value: ns > 0.05, * ≤0.05, ** ≤0.01, *** ≤0.001, **** ≤0.0001.

We established that at least one mutation in each of the eight G-runs capable of contributing to G-tetrad formation at the *MYC* G4 site was required to prevent G4 folding in vitro (*SI Appendix*, *Supporting Text*). A mutant, designated MYC MUT, was chosen to ensure G4 structure formation was completely abrogated, as assessed both by biophysical measurements *SI Appendix*, Figs. S1 and S2) and the absence of G4 formation in cells (Figs. 1 and 2). Cis-BP scoring confirmed that the introduced mutations did not disrupt any critical TF binding motifs.

Another perturbation was generated by removing the endogenous MYC WT sequence and replacing it with a different G4 sequence motif from the *KRAS* locus, designated KRAS SWP (Fig. 1*B*). The *KRAS* G4 has been the subject of detailed biophysical and structural studies, and like the *MYC* G4, it forms a parallel G4 structure, albeit with longer loops (44–46). Comparison of the edited sites, by imposing penalties for mismatches and gaps, showed that the MYC MUT sequence was closely related to MYC WT, whereas KRAS SWP was substantially dissimilar (*SI Appendix*, Fig. S3). Several clones for each edited cell line were expanded and confirmed to have the desired edits by Sanger sequencing across the targeted region (*SI Appendix*, Fig. S4).

G4 formation in the MYC WT and KRAS SWP oligonucleotide sequences and the absence of G4 formation for the MYC MUT sequence were confirmed in vitro using biophysical measurements. CD spectroscopy showed that the MYC WT and KRAS SWP have signatures characteristic of parallel G4 formation (maxima ~263 nm, minima ~245 nm) (31, 35), whereas the MYC MUT spectrum (maxima ~280 nm, minima ~250 nm) was consistent with the absence of G4 folding (Fig. 2*A*). CD thermal melting spectroscopy shows that the *MYC* G4 (T_m_ = 69 °C) was more stable than *KRAS* G4 (T_m_ = 52 °C, Fig. 2*B*).

We next investigated whether folded G4 structures formed at the *MYC* locus in the WT and edited cells. Topologically, G4 structure formation would lead to proximal single-strandedness; therefore, we used Kethoxal-assisted single-stranded-qPCR [KAS-qPCR, (47)] to assess single-strandedness at the *MYC* promoter locus. Consistent with a transition from single-strand towards duplex, we observed a decrease in the KAS-qPCR signal at MYC MUT relative to MYC WT (*P*-value = 0.04, Fig. 2*C*). Next, we used three distinct G4 structure-specific probes to evaluate folded G4 formation. Using G4 CUT&Tag with BG4, a well-characterized G4-structure-specific scFv antibody, we observed a drop in G4 signal in the MYC MUT compared to MYC WT, with the G4 signal being restored in the KRAS SWP cells (Fig. 2 *D* and *E*). Using targeted BG4 CUT&Tag-qPCR, we quantified the G4 signal relative to unaltered G4s in three control genes (*RPA3, MAZ, RBBP4*) and found a ~78% loss in G4 formation at MYC MUT (*P*-value = 0.033, *P*-value = 0.0009, and *P*-value = 0.005 when calculated against controls *RPA3, MAZ,* and *RBBP4* G4s, respectively, Fig. 2*E* and *SI Appendix*, Table S1) and ~74% recovery of G4 formation (*P*-value = 0.0199, *P*-value = 0.0073, and *P*-value = 0.1295 when calculated against *RPA3, MAZ,* and *RBBP4* G4s, respectively) with KRAS SWP. Using our recently developed Chem-map approach (24), we measured the chromatin binding of a G4-specific small molecule, pyridostatin [PDS, (8)], to further validate loss of G4 formation (~90%, *P*-value < 0.0001) in MYC MUT cells and recovery of G4 signal (~75%) in KRAS SWP cells (*P*-value = 0.0058, *P*-value = 0.0119, and *P*-value = 0.1391 when calculated against *RPA3*, *MAZ,* and *RBBP4* G4s, respectively, Fig. 2*F* and *SI Appendix*, Table S1). Last, we utilized the recently developed SG4 nanobody, which was generated by affinity selection against the *MYC* G4 structure, alongside a SG4 mutated control nanobody that is unable to bind G4s (12). The SG4 CUT&Tag experiments confirmed the loss of G4 signal in MYC MUT (~83%, *P*-value < 0.0001, *P*-value = 0.0074, and *P*-value = 0.005 when calculated against *RPA3*, *MAZ,* and *RBBP4* G4s, respectively, *SI Appendix*, Table S1) and recovery of signal in KRAS SWP cells (~92%, *P*-value = 0.1324, *P*-value = 0.0241, and *P*-value = 0.4415 when calculated against *RPA3*, *MAZ,* and *RBBP4* G4s, respectively, Fig. 2*G* and *SI Appendix*, Table S1). Taken together, these multiple lines of data verify that in cells we can site-specifically remove the folded *MYC* G4 structure by mutagenesis and restore a G4 structure by substitution with a different G4-forming DNA sequence.

### G4 Structure Positively Regulates *MYC* Transcription and Controls P1 Promoter Activity.

**Fig. 3.**
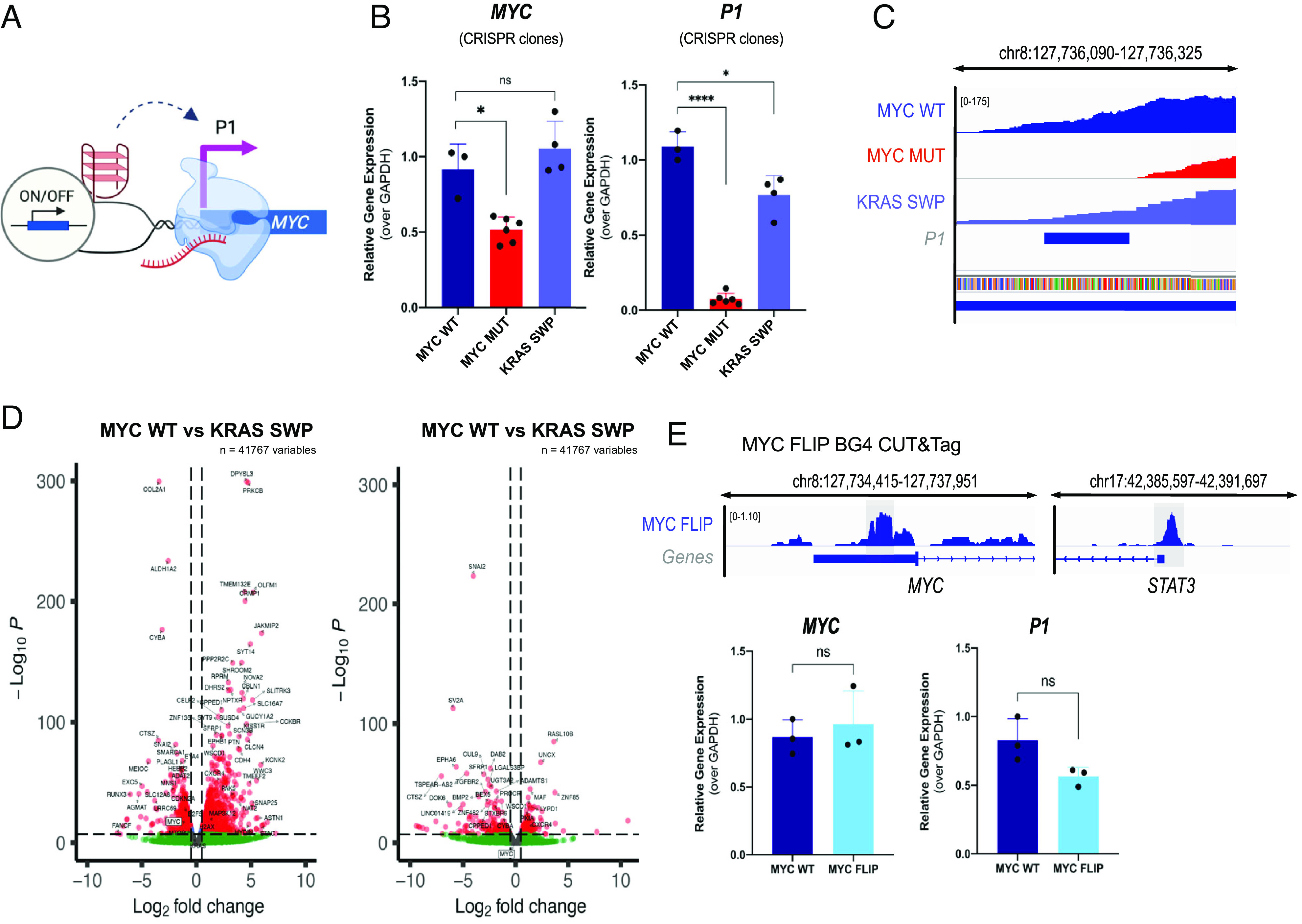
Loss of the *MYC* G4 structure abolishes *MYC* transcription from promoter P1. (*A*) Schematic of the G4 position in the upstream regulatory region of the *MYC* promoter P1. (*B*) RT-qPCR data for total *MYC* expression and P1 expression for multiple clones for each CRISPR-derived edit. Mean ± SD of MYC MUT (N = 3), KRAS SWP (N = 2) independent clones, for n = 2 biological replicates, measured as technical triplicates. (*C*) RNA-seq tracks (IGV) at the *MYC* P1 promoter site showing the absence of transcripts in MYC MUT cells compared to MYC WT and KRAS SWP cells. Blue bar indicates the P1 amplified region. (*D*) Volcano plots of RNA-seq data (*P*adj = 0.05, Log2 FC = 0.5). (*E*) Genomic binding profile (IGV track) showing BG4 CUT&Tag for the MYC FLIP cell line (*Top*). Gray regions indicate the location of G4 formation. RT-qPCR data for total *MYC* and P1 expression for the MYC FLIP genetic edit (*Bottom*). Mean ± SD, n = 3 independent biological samples. *P*-value: ns > 0.05, * ≤0.05, ** ≤0.01, *** ≤0.001, **** ≤0.0001.

Given the association between G4s and transcription, and the observation that some TFs can bind folded G4s (1, 18), we next measured whether G4 loss alters *MYC* expression (Fig. 3*A*). We observed a significant reduction in total *MYC* RNA levels across the clonal population upon G4 loss, as revealed by RT-qPCR (44% reduction relative to MYC WT, *P*-value = 0.0417, Fig. 3*B*) and RNA-seq (51% reduction, q-value = 9.00 E-15, Fig. 3 *C* and *D*). Accordingly, in MYC MUT cells, we observed that total MYC protein was reduced by 38% (*P*-value = 0.0173), as assessed by western blot (*SI Appendix*, Fig. S5). MYC protein and RNA levels were restored in KRAS SWP cells (90% protein, *P*-value = 0.0411 by western blot; 97% RNA, q-value = 0.97 as determined by RNA-seq) (Fig. 3 *B*–*D* and *SI Appendix*, Fig. S5). In MYC MUT cells, P1-driven transcription was completely abolished relative to MYC WT, as verified by RNA-seq (q-value = 4.53E-27) and RT-qPCR (~99% reduction, *P*-value < 0.0001, Fig. 3 *B* and *C*). Conversely, adding back a different folded G4 in KRAS SWP cells restored P1 promoter transcription to near-WT levels (96% by RNA-seq, and 70% as determined by qPCR, Fig. 3 *B* and *C* and *SI Appendix*, Table S2). Total MYC levels were also recovered in KRAS SWP clones, as assessed by RT-qPCR (100%, *P*-value = 0.3449). These results were further validated with an independent CRISPR edit, designated MUT MIN, that abrogated *MYC G4* structure with different sequence mutations (*SI Appendix*, Fig. S6).

We probed whether G4 strandedness was important in this context, by moving the natural G4 motif sequence across to the template rather than non-template strand of MYC WT to generate MYC FLIP (Fig. 1*B*). Moving the G4 to the opposite strand does not have a significant effect on *MYC* or P1 promoter expression (*P*-value = 0.5881, *P*-value = 0.0899, respectively, Fig. 3*E*).

Overall, these data show that a folded G4 structure, rather than sequence per se, augments *MYC* expression from the P1 promoter. Ontology analysis also shows the downstream events caused by loss of *MYC* G4. Reduction in *MYC* expression leads to down-regulation of known MYC targets, mRNA splicing and translation pathways, genes involved in cell division, proliferation, genomic instability, and hallmarks of cancer (*SI Appendix*, Figs. S7–S10).

### TF Recruitment to the *MYC* Promoter Requires G4 Structure Formation.

**Fig. 4.**
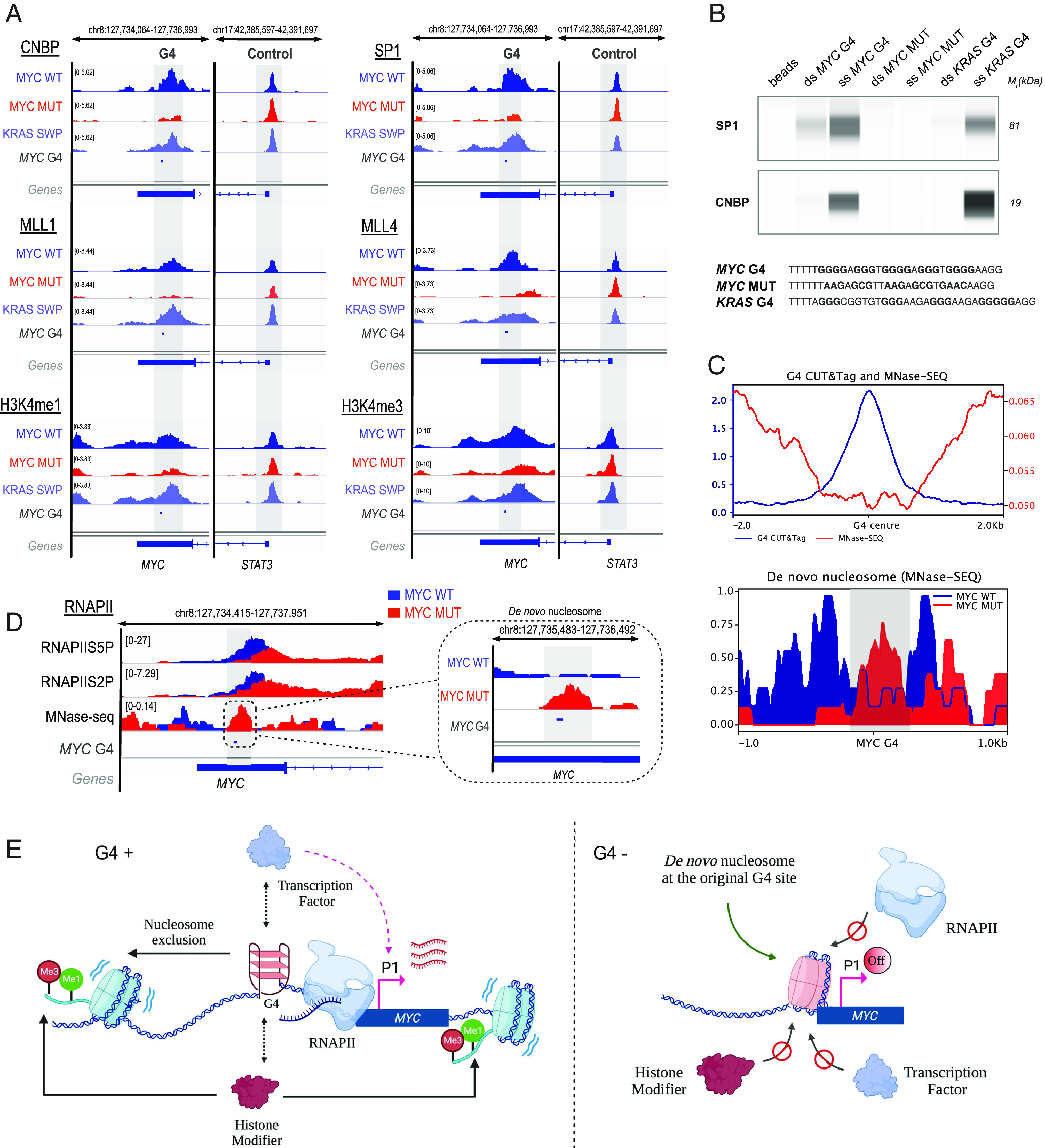
The *MYC* G4 structure organizes the local chromatin landscape. (*A*) Genomic CUT&Tag binding profiles (IGV tracks) for SP1 and CNBP TFs (*Top*), MLL1 and 4 methyltransferases (*Middle*), and histone H3K4me1 and H3K4me3 methylation (*Bottom*) in MYC WT, MYC MUT, and KRAS SWP cells. (*B*) Affinity enrichment and western blot analysis for SP1 and CNBP proteins for double-strand (ds) and single-strand (ss) *MYC* G4, ss/ds *MYC* MUT, and ss/ds *KRAS* G4. (*C*) Genome-wide distribution of G4 CUT&Tag and MNase-seq data (*Top*). MNase-seq profile illustrating the deposition of a de novo nucleosome at the edited site (*Bottom*). (*D*) Genomic binding profiles (IGV tracks) showing RNAPII CUT&Tag for transcriptional initiation (RNAPIIS5P) and elongation (RNAPIIS2P), as well as nucleosome positioning (MNase-seq). (*E*) Suggested model where the G4 plays a central role to orchestrate a series of transcriptional events, including TF binding, molding the epigenetic landscape, organizing nucleosomes, and positioning RNAPII to regulate transcription in *MYC*.

We then explored how loss of the folded *MYC* G4 alters the recruitment of SP1 and CNBP TFs, each known to bind folded G4s from biophysical studies and thought to be involved in *MYC* transcription (48, 49). Chromatin profiling of each TF using CUT&Tag showed that at the edited *MYC* locus both SP1 and CNBP are only recruited in the presence of a folded G4 structure (Fig. 4*A*). Changes in SP1 and CNBP profiles at other binding sites genome-wide were not evident between MYC WT, MYC MUT, and KRAS SWP (*SI Appendix*, Figs. S11 and S12). To rule out that the introduced mutations affect TF binding primarily via altered interactions with the natural duplex DNA sequence, we carried out affinity enrichment of proteins from nuclear extracts using folded G4 oligonucleotides for *MYC* and *KRAS* and their corresponding duplex and mutant sequence controls. Both SP1 and CNBP bind to the folded G4 structures formed by *MYC* and *KRAS*, whereas each TF exhibited weaker binding to the corresponding double-stranded oligonucleotides (MYC WT, KRAS SWP, Fig. 4*B*). Furthermore, neither SP1 nor CNBP exhibited binding to the mutated single-stranded or double-stranded MYC MUT DNA sequence (Fig. 4*B*). These data support that in cells the folded G4 structure in the *MYC* promoter, rather than its duplex counterpart, is key for TF engagement and that it is the loss of folded G4 structure in the mutant (MYC MUT) that causes loss of TF binding. The absence of strong SP1 binding to the MYC WT duplex DNA (Fig. 4*B*) is consistent with the Pu27 sequence not containing an SP1 consensus site, with the closest one being 71 bp downstream in a region unaffected by the genome editing. Biolayer interferometry analysis further confirmed selective and direct SP1 binding to the single-stranded *MYC* G4 (K_D_ = 5.6 nM, *SI Appendix*, Fig. S13*A*) with substantially weaker binding to the corresponding duplex DNA (K_D_ = 252 nM, *SI Appendix*, Fig. S13 *B*, *C*) and negligible binding to single-stranded MYC MUT (*SI Appendix*, Fig. S13*D*). Thus, transcriptional activation of the *MYC* P1 promoter depends on a folded G4 structure that forms within the *MYC* NHE III_1_ regulatory element to recruit the appropriate TFs.

### *MYC* G4 Loss Alters the Local Chromatin Landscape.

We next measured how loss of the folded G4 alters the chromatin landscape. Using MNase-seq, we mapped nucleosomes in HEK293T cells across the genome. Globally, the genome-wide distribution of G4s and nucleosome occupancy showed an inverse relationship consistent with G4 structure formation being incompatible with the presence of a nucleosome (Fig. 4*C*). We then interrogated nucleosome positioning in MYC MUT cells by MNase-seq. Importantly, G4 loss led to the de novo deposition of a nucleosome at the edited site (Fig. 4*C* and *SI Appendix*, Fig. S14). These results show that the folded *MYC* G4 has a key role in coordinating nucleosome positioning by promoting nucleosome exclusion.

We next investigated the histone landscape. G4s have previously been implicated in histone methylation in promoters (50) and in the recruitment of histone modifiers such as histone methyltransferases (18). Furthermore, we previously observed a link between G4s and chromatin remodeling proteins in functional genomic screens for synthetic lethality (51). Mixed Lineage Leukemia 1 (MLL1/KMT2A) and 4 (MLL4/KMT2D) methylate histone 3 lysine 4 (H3K4) and localize at promoters to promote transcriptional activation (52, 53). Genome-wide, we find that MLL1 binding and active H3K4me1/3 methylation, but not repressive H3K27me3, also overlap at sites where G4 structures are detected (*SI Appendix*, Fig. S15). Binding of both MLL1 and MLL4 is depleted at the G4-edited site in MYC MUT compared to MYC WT cells, whereas binding is recovered in KRAS SWP cells (Fig. 4*A* and *SI Appendix*, Table S3). Affinity pull-down from nuclear lysates provides additional support for selective binding of MLL4 to folded G4 DNA structures (*SI Appendix*, Fig. S16). The active histone methylation marks H3K4me1 and H3K4me3, normally installed by MLL1 and MLL4, were lost when the folded G4 is removed in MYC MUT cells (Fig. 4*A*). Thus, the folded *MYC* G4 structure influences the local H3K4 methylome via a process that involves recruitment of histone modifiers.

### RNA Polymerase II Positioning by the *MYC* G4.

Given that *MYC* G4 loss abrogates *MYC* transcription from the P1 promoter, we investigated whether this arises through alterations in RNA polymerase II (RNAPII) engagement. We mapped initiating (RNAPIIS5P) and elongating RNA polymerase II (RNAPIIS2P) in MYC WT, MYC MUT, and KRAS SWP cells. In MYC WT cells, RNAPIIS5P and RNAPIIS2P are located immediately downstream of the *MYC* G4; however, in the absence of the G4 structure, both RNAPIIS5P and RNAPIIS2P showed a statistically significant drop in occupancy (55.73%, *P*-value = 0.0042 and 41.88%, *P*-value = 0.0095, respectively). Moreover, the RNAPII CUT&Tag signal maxima were displaced 261 bp toward the 3′ end (Fig. 4*D*). KRAS SWP cells showed RNAPII profiles comparable to MYC WT cells (*SI Appendix*, Fig. S17*A*). We then compared how alterations in nucleosome occupancy upon G4 loss were related to RNAPII engagement (Fig. 4*D*). By comparing MNase-seq and RNAPII CUT&Tag data, we observed that the deposition of a new nucleosome upon G4 loss is accompanied by the loss of RNA polymerase engagement at the edited site. These data suggest that *MYC* P1 transcriptional activity depends on a G4 structure to prevent deposition of a nucleosome that would otherwise impede RNA polymerase progression.

As changes in the RNAPII chromatin profiles were detected, we interrogated R-loop formation in edited and WT cells using CUT&Tag and observed no statistically significant changes in R-loop signal at the *MYC* locus when comparing MYC WT to MYC MUT (*P*-value = 0.4378) and KRAS SWP (*P*-value = 0.9603) (*SI Appendix*, Fig. S17*B*). This suggests that folded G4 structure does not affect R-loop formation in this context. Furthermore, to specifically address whether transcription promotes G4 folding at *MYC*, we inhibited transcriptional initiation and elongation in MYC WT cells by treatment with triptolide (54), or flavopiridol (55), respectively. In both cases, we observed no changes to G4 levels (*SI Appendix*, Figs. S18 and S19), which suggests that active transcription is not required to maintain G4 folding at the *MYC* locus. This is consistent with earlier observations demonstrating that active transcription is not necessarily required for G4 folding (22). Consequently, the engagement and positioning of RNA polymerase II are regulated by the *MYC* G4 structure, with direct effects to transcriptional output (Fig. 4*E*).

## Discussion

Recent advancements in the detection of folded G4 structures in chromatin and in genome manipulation have allowed us to probe the structure and function of a natural G4 in a cellular, genomic context, which was previously not possible. Here, we have focused on a single, folded G4 within the context of human *MYC* NHE III_1_. Through multiple independent approaches, we confirmed G4 folding in cells and the loss of folded G4 upon mutation of critical G bases. For this G4, it is imperative to consider proximal G runs outside of the core Pu27 sequence that can contribute to G4 folding. Some prior studies used a shorter sequence, such as Pu27 alone, which does not reflect the full G4 forming potential of the eight G-runs in the *MYC* NHE III_1_. Earlier studies that investigated the *MYC* G4 in vitro or in plasmids may be missing the critical contributions of the chromatin context.

Our findings highlight several facets of how the *MYC* G4 structure can facilitate transcription. The data suggest that the *MYC* G4 has a primary role in nucleosome positioning, facilitating chromatin accessibility and preventing nucleosome deposition that would otherwise impede RNAPII engagement. G4 loss leads to a reduction in RNAPII occupancy, whereas the loss of RNAPII activity does not affect G4 folding. G4-mediated deposition of active histone marks (H3K4me1, H3K4me3) by histone methyltransferases (MLL1 and MLL4) reinforces the role of G4 structures in priming chromatin for transcription. Moreover, our work, showing that loss of the *MYC* G4 leads to the loss of TF binding in cells, builds on earlier studies that demonstrate the potential for TFs to bind G4 structures (1, 18). This supports the functional role of a G4 acting as a positive regulator of *MYC* transcription. The *KRAS* and *MYC* G4s are both parallel structures, though the *KRAS* G4 has longer loops. Given that the *KRAS* G4 can functionally substitute for the *MYC* G4, this suggests that interacting proteins recognize shared features such as the π-rich, terminal G-tetrads, and the high negative charge density in the G4s. We also demonstrate that the *MYC* G4 structure can mold the local epigenetic states and dictate chromatin architecture.

To further understand the importance of this G4 structure, we examined conservation of the *MYC* promoter G4 across different species. Those closely related to humans, such as gorillas, bonobos, and chimpanzees have almost complete conservation of the Pu27 element and flanking sequences (*SI Appendix*, Fig. S20*A*). While other mammalian species, such as mouse, show less sequence conservation, the region still comprises a folded G4 structure as shown by G4 CUT&Tag (*SI Appendix*, Fig. S20*B*). In contrast, other vertebrates, such as zebrafish, show little sequence conservation suggesting that mammals have evolved a unique *MYC* regulatory module that, in part, is based on G4 secondary structures.

Overall, we conclude that G4 structures orchestrate a series of transcriptional events. The evidence suggests a mechanism of action whereby a G4 structure mediates TF binding, alters the histone methylome, coordinates nucleosome occupancy, and positions RNA polymerase II to modulate *MYC* transcription. These results suggest a critical role of G4s in regulating gene expression, whereby DNA structure, rather than sequence is key. The use of small molecules to stabilize G4s should also be considered carefully. G4 ligands can compete for TF binding to G4s (18), thus explaining transcription inhibition. Our results now show that a natural G4 normally promotes transcription thus interventions that stabilize G4s might alternatively result in enhanced transcription. Given the enrichment of G4 sequence motifs and detection of folded G4 structures in gene promoters throughout the genome, we anticipate that other G4 structures will have an active involvement in transcriptional regulation in other mammalian genes.

## Materials and Methods

### CRISPR/Cas9 Design.

The upstream regulatory region sequence containing the G4 of the human *MYC* locus was extracted using the UCSC genome browser. Guide RNA targeting sequences specific to this locus, and on-target efficiencies were assessed using CRISPOR (http://crispor.tefor.net/crispor.py). Different sgRNA efficiencies were tested using a T7E1 assay (New England Biolabs, cat #E3321S) and the most efficient guide selected for editing (*SI Appendix*, Table S4). Homology repair templates (HRT) were designed to target the edited site (chr8: (-) 127,735,928-127,735,954) on the DNA leading strand, including upstream and downstream flanking regions, to a total of 200 bp (*SI Appendix*, Table S5). Genotyping was performed by amplicon Sanger sequencing (*SI Appendix*, Table S6). CRISPR editing was performed by plasmid transfection or electroporation (see *SI Appendix* for further details and genotyping strategy).

### Cell Culture.

Human embryonic kidney (HEK293T) cells were cultured in DMEM (Dulbecco‘s Modified Eagle Medium, Gibco™, cat #41966-029) growth media supplemented with 10% fetal bovine serum (Gibco™, A3840401) and 2 mM L-glutamine (Gibco™, cat #25030024). Cells were maintained under standard conditions (5% CO_2_, 37 °C) on 6-well plates (Corning, cat #3516) and regularly tested for mycoplasma contamination. Cells were authenticated using human STR analysis by Research Instrumentation and Cell Services (RICS), CRUK Cambridge Institute. See *SI Appendix* for further cell culture details.

### Cellular Transfection, FACS Sorting.

For transfection-based editing, cells were seeded 24 h prior transfection. Transfections were performed at 60 to 80% confluency using Lipofectamine LTX PLUS (ThermoFisher Scientific, cat #15338100). Plasmid DNA with Cas9 endonuclease and the relevant sgRNA cloned into the GFP-containing backbone plasmid (Addgene, cat #48138) were used for expression. Then, 48 h post transfection, cells were washed with DPBS pH 7.4 (Gibco™, cat #14190094) and dissociated with StemPro™ Accutase™ (Gibco™, cat #A1110501) at 37 °C for 5 min. The resulting pellet was resuspended in fresh media and passed through a cell strainer snap cap (Corning, cat #352235). Positively transfected cells (GFP-positive) were subcloned into 96-well plates (Corning, cat #3595). Cell sorting was performed using purity mode at a flow rate of 100 on a fluorescence-activated cell sorter (FACS) Melody™ instrument (Beckmann-Dickinson) with stringent gating for the top 60% of the positive parent population (*SI Appendix*, Fig. S21). Electroporated cells were subcloned into 96-well plates using the same sorting parameters on a FACS Aria II™ instrument (Beckmann-Dickinson).

### Transcriptional Inhibitor Treatment.

WT HEK293T cells were grown on 6-well plates until 80 to 90% confluent. Cells were treated with triptolide (Cayman Chemicals, cat #11973-1 mg-CAY, 10 mM in DMSO) or flavopiridol hydrochloride (Cayman Chemicals, cat #10009197-5 mg-CAY, 1 mM in DMSO) a final concentration of 10 μM or 1 μM, respectively, in complete growth medium for 30 min, 1 h, and 2 h. As vehicle control, cells were treated with DMSO (diluted 1:1,000 v/v in complete medium) for 2 h. Cells were harvested using StemPro™ Accutase™ (Gibco™, cat #A1110501), resuspended in cold medium and kept on ice until further processing with CUT&Tag.

### BG4 scFv and SG4 Antibodies.

The expression vectors pSAN10-3F-BG4 (Addgene, cat #55756) and pHEN2-SG4 (Addgene, cat #196071) were used to for G-quadruplex structure-specific antibody expression. BG4 (scFv antibody) and SG4 (nanobody) were expressed, purified, and validated as previously described (9, 12).

### Real-Time Quantitative PCR.

RNA from three biological replicates (1.0 × 10^6^ cells each) was reverse transcribed to complementary DNA (cDNA) using the iScript™ cDNA Synthesis Kit (Bio-Rad, cat #1708890). For cDNA synthesis, 1 μg of RNA was used. qPCR reactions were prepared as follows: cDNA was mixed with the relevant primer mix (*SI Appendix*, Table S7, 500 nM) in iTaq™ Universal SYBR Green (Bio-Rad, cat #1725120). Three technical replicates were prepared per sample and qPCR performed on a 96-well plate format using a QuantStudio5 Real-Time PCR system (ThermoFisher Scientific) with the following program: 95 °C for 3 min, 40× (95 °C for 15 s, 56 °C for 30 s, 72 °C for 30 s), 95 °C for 15 s, 60 °C for 1 min, and 95 °C for 15 s (continuous melting curve). C_t_ values were extracted using QuantStudio design and analysis software (v1.5.1) and relative fold-change in gene expression calculated using the ΔΔC_t_ method normalized to *GAPDH*. Statistical significance was calculated with the Welch-corrected, two-tailed, Student *t*-test. Data are from means ± SD (n = 3), with ns: not significant; *P ≤ 0.05, **P ≤ 0.01, ***P ≤ 0.001, ****P ≤ 0.0001.

### CD Spectroscopy and Thermal Melting Assay.

CD measurements were collected on an Applied Photophysics Chirascan spectropolarimeter using an optical path length of 1 mm. Oligonucleotide solutions (*SI Appendix*, Table S8) in 20 mM lithium cacodylate (pH 7.0) containing 10 or 100 mM KCl or LiCl (56) were annealed by heating at 95 °C for 5 min followed by slow cooling to room temperature. Scans were performed over the range of 210 to 330 nm at 20 °C in triplicate. Thermal melting was performed using a quartz cuvette with a 1-cm path length. Samples, covered with a layer of mineral oil, were heated from 20 to 93 °C. Measurements were monitored consecutively at a rate of 1 °C/min at 263 nm and data collected in triplicate every 1 °C step. Data points were fitted to an asymmetric sigmoidal curve (5PL) using GraphPad Prism (Version 8.3.0). T_m_ values were calculated with Van’t Hoff analysis, where the T_m_ is the temperature at which the folded fraction (θ) equals 0.5 (57).

### UV Spectroscopy and Thermal Melting Assay.

Oligonucleotides (*SI Appendix*, Table S8) in 20 mM lithium cacodylate (pH 7.0) containing 10 or 100 mM KCl or LiCl (56) were annealed at 95 °C for 5 min followed by slow cooling to room temperature. UV spectral absorbances were measured between 200 and 330 nm using an Agilent Cary 3500 Multicell UV–Vis spectrophotometer. Measurements were made using a 1-cm path length quartz cuvette covered with a layer of mineral oil. Thermal differences were calculated by subtraction of the 20 and 90 °C spectra. Samples were heated from 20 to 90 °C and UV melting curves were recorded at a spectral bandwidth of 2 nm, with the temperature measured consecutively at a rate of 0.5 °C/min at 300 and 305 nm, with data collection in triplicate every 0.2 °C. T_m_ values were obtained from Van’t Hoff analysis of the melting profiles as the temperature at which the folded fraction (θ) equals 0.5 (57).

### Cleavage Under Targets and Tagmentation (CUT&Tag) and Chem-map.

CUT&Tag and Chem-map were performed as previously described (24, 58), with minor modifications (see *SI Appendix* for further details). Cells or nuclei were fixed and immobilized on ConA-conjugated magnetic beads. Primary antibodies or biotinylated PDS (*SI Appendix*, Table S9) were added (1:50, 400 nM PDS), followed by incubation at 4 °C overnight at 600 rpm. Secondary antibodies (*SI Appendix*, Table S10) were added (1:50) and samples incubated at 25 °C for 2 h at 600 rpm (2-layer CUT&Tag). For G4-mapping samples, tertiary antibody (1:100) was added followed by pA-Tn5 (1:250) and samples incubated at 25 °C for 1 h at 600 rpm. Tagmented DNA was purified with DNA clean & concentrator (Zymo Research, cat #D4013) Sequencing libraries were generated as previously described using universal i5 and uniquely barcoded i7 primers (24, 58). Sequencing was performed on an Illumina NextSeq 500 (High Output Kit v2.5, 75 cycles) or NextSeq 2000 instrument (P2 or P3 reagents, 100 cycles). See *SI Appendix* for sequencing data processing.

### CUT&Tag and Chem-map-qPCR.

Genomic DNA isolated from CUT&Tag and Chem-map samples was used to quantify the relative fold signal at the G4 target site using gene-specific primers for 3 G4 positive sites (*RPA3, MAZ, RBBP4*) (59). See *SI Appendix* for *MYC* primer design and qPCR analysis. First, 2.5 µL CUT&Tag library was diluted to 1:15 to 1:30 and added to 2.5 µL [1 µM] primer mix and 5 µL SYBR Green PCR Master Mix (Applied Biosystems; ThermoFisher Scientific, cat #4312704). The following program was run: 20 s at 95 °C (10 s at 95 °C, 30 s at 60 °C, 10 s at 72 °C), for 39 cycles before heating to 90 °C. Then, data were analyzed by the ΔΔC_t_ method for the target site against three G4 positive sites. Statistical significance was calculated on the resulting normalized fold-changes with the Welch-corrected, two-tailed, Student *t*-test. Data are from means ± SD (n = 6), with ns: not significant; *P ≤ 0.05. See *SI Appendix*, Table S11 for primer sequences.

### MNase-seq and Library Preparation.

First, 1.0 × 10^6^ cells washed in ice-cold PBS were incubated in 1 mL lysis buffer (10 mM Tris-HCl pH 8.0, 60 mM KCl, 15 mM NaCl, 3 mM MgCl_2_, 1 mM DTT, 0.1% NP-40) for 10 min on ice. Then, cells were centrifuged at 1,000×*g* for 5 min at 4 °C and resuspended in lysis buffer without NP-40 (10 mM Tris-HCl pH 8.0, 60 mM KCl, 15 mM NaCl, 3 mM MgCl_2_, and 1 mM DTT). Cells were then incubated at 37 °C for 5 min supplemented with 2 mM CaCl_2_ and digested with 10 U MNase at 37 °C for 20, 40, and 60 min. MNase digestion was stopped with a final concentration of 40 mM EDTA, 0.5% SDS followed by RNase A (ThermoFisher Scientific, cat #EN0531) treatment for 1 h at 37 °C. DNA was purified using Phenol-chloroform-isoamyl alcohol (PCl, ThermoFisher Scientific, cat #15593049). Libraries were generated with the NEBNext® Ultra^TM^ II Directional RNA Library Prep Kit for Illumina (New England Biolabs, cat #7760S). Bead-based size selection for 200 bp was performed to isolate mononucleosomal fragments from the 60 min digestion point. Libraries were quantified with the NEBNext® Library Quant Kit for Illumina (New England Biolabs, cat #7630S) and sequenced on a NextSeq 500 system (High Output Kit v2.5, 75 cycles).

### RNA-seq and Library Preparation.

Total RNA was extracted from 1.0 × 10^6^ cells using the RNeasy Plus Mini Kit (Qiagen, cat #74134). RNA quality was checked via TapeStation (Agilent Technologies 2200, RIN > 9) and RNA concentration determined using a Qubit4 fluorometer (ThermoFisher Scientific). Libraries were generated from 700 ng RNA using the NEBNext® Poly(A) mRNA Magnetic Isolation Module (New England Biolabs, cat #7490S) together with the NEBNext® Ultra^TM^ II Directional RNA Library Prep Kit for Illumina (New England Biolabs, cat #7760S). Libraries were sequenced on a NextSeq 500 system (High Output Kit v2.5, 75 cycles). See *SI Appendix* for Data Processing details.

### G4 Affinity Enrichment and Analysis.

Oligonucleotides (PAGE-purified) were obtained from Sigma-Aldrich or Integrated DNA Technologies. For G4 formation, 10 mM oligonucleotide was annealed in 10 mM Tris-HCl, pH 7.4, and 100 mM KCl by heating at 95 °C for 5 min followed by gradual cooling to 20 °C. For double-stranded DNA, 10 mM top and bottom strands were mixed and annealed in 10 mM Tris-HCl, pH 7.4. G4 pull-down and subsequent protein analysis by western blotting was performed using a WES Protein Simple instrument as described previously (18, 19). See *SI Appendix*, Table S12 for oligonucleotide sequences.

### N3-Kethoxal Assisted-ssDNA (KAS)-qPCR.

KAS labeling was performed using 1.5 × 10^6^ HEK293T cells as described in ref. 47. To perform KAS-qPCR, eluted DNA was diluted 1:4.5 and input (sonicated but not biotin-enriched) samples were diluted 1:7 in nuclease-free water (Ambion, cat #AM9937). qPCRs were set up with 5 μL Fast SYBR Green Master Mix (ThermoFisher Scientific, cat #4385612), 2.5 μL diluted sample and 2.5 μL primer mix at 1 μM and performed on a C1000 Touch CFX384 thermal cycler (Bio-Rad) with the following program: 95 °C for 20 s, 95 °C for 10 s, 59 °C for 30 s and 72 °C for 10 s, with steps 2 to 4 repeated for a total of 40 cycles. See *SI Appendix*, Table S13 for primer sequences. Melting curves were measured using 0.5 °C s^−1^ steps from 65 to 95 °C. Three technical replicates were performed for each reaction. Input and sample Ct values were adjusted to 100% (log2 of dilution factor). % Input was calculated as 100 × 2^(average input Ct − average sample Ct)^. Calculated % input values and statistical analyses are in *SI Appendix*, Table S14.

### Western Blot Analysis.

For protein analysis 1.0 × 10^5^ cells were plated in a 60-mm dish for 48 h. 5.0 × 10^5^ cells each were harvested for analysis in 60 μL Pierce^TM^ RIPA buffer (ThermoFisher Scientific, cat #89901) supplemented with 1× Halt Protease Inhibitor Cocktail (ThermoFisher Scientific, cat #79438). Cell lysates were incubated on ice for 15 min and then sonicated for two cycles (30 s on/60 s off, high mode) using a water-cooled bath sonicator (Diagenode, Bioruptor Plus) and centrifuged at 21,000×*g* at 4 °C for 30 min. Protein concentration was assessed by Direct Detect Spectrometer (Merck). Capillary electrophoresis on a WES Protein Simple Instrument was performed according (final protein conc. of 1.5 mg/mL) using an anti-rabbit detection module (25-capillary 12-230 kDa; ProteinSimple, cat #SM-W004 and detection kit (ProteinSimple, cat #DM-001) and corresponding anti-rabbit primary antibodies: c-Myc, 1:1,000 (Cell Signaling Technology, RRID: AB_1903938, cat #CS5605S) and β-Actin, 1:2,000 (Cell Signaling Technology, RRID: AB_2223172, cat #CS4970S). Protein bands were visualized using Compass Software (ProteinSimple) and the drop in intensity estimated from the area-under-the-curve (AUC). Statistical testing was performed using the Wilcoxon rank-sum exact test for non-normally distributed data. *P*-values were obtained from the R base implementation to examine whether relative, normalized signals were reduced in MYC MUT vs. WT. Data are from means ± SD (n = 3), with ns: not significant; *P ≤ 0.05.

### Biolayer Interferometry (BLI).

BLI experiments were performed using an Octet RED96 instrument. First, 100 nM solutions of biotinylated single-stranded and double-stranded oligonucleotides were incubated with streptavidin biosensors in 25 mM HEPES pH 7.5 12.5 mM MgCl_2_, 20% v/v glycerol, 0.1% v/v Igepal CA-630, 0.01 mM Zn(OAc)_2_, 1 mM dithiothreitol, and 100 mM KCl 3% BSA. Then, to compare binding profiles (*SI Appendix*, Table S13 and Fig. S13), 100 nM of recombinant SP1 protein (full-length human sequence, Active Motif, cat #81181), an oligo loading time of 120 s and an association/dissociation time of 1,000 s were used. For the full single-stranded *MYC* G4 assay titration of different concentrations of SP1, an oligo loading time of 240 s and an association/dissociation time of 1,200 s were used. For the full double-stranded *MYC* titration, 120-s loading time and 1,000-s association/dissociation time were used. Binding curves obtained from both titrations were fitted using a 2:1 heterogeneous ligand model with Savitzky–Golay filtering.

### Identification of TF Binding Motifs in Targeted Sequences.

Different DNA sequences were scanned for the presence of TF binding motifs and binding preferences (sequencing) using data from Cis-BP database (v.2.0) (40), a catalog of direct and inferred sequence binding TFs preferences from 290 eukaryotic genomes. Each individual DNA sequence was scanned against the mouse specific TFs Position Weight Matrices (PWMs) using the Cis-BP built-in tool. The output contains information on binding of putative TFs, strand of the putative match for the motif (mathematically defined with PWMs) in the query DNA sequence and a score for the putative binding location. The score represents the log-odds of the TF motif preference (binding, minimum 8-mer binding) and approximates (simplifying) the free energy of TF-DNA binding to the original sequence.

### Motif Analysis.

Consensus peaks from CUT&Tag experiments (common among two out of three biological replicates) for SP1 and CNBP were imported into XSTREME (meme-suit v5.4.1) for motif identification analysis. The results show centrally localized motifs due to SP1 and CNBP binding (*SI Appendix*, Fig. S12).

## Supplementary Material

Appendix 01 (PDF)

## Data Availability

Sequencing data generated in this study have been submitted to the NCBI Gene Expression Omnibus (GEO; https://www.ncbi.nlm.nih.gov/geo/) under accession number GSE223370 (60). All other data are included in the manuscript and/or *SI Appendix*.
