# Supplementary material for: G-quadruplex DNA structure is a positive regulator of MYC transcription
Source: Proc Natl Acad Sci U S A. 2024 Feb 5;121(7):e2320240121. doi: 10.1073/pnas.2320240121 (PMC10873556; doi:10.1073/pnas.2320240121)
Supplement: Supplementary file 1 — Appendix 01 (PDF) [file pnas.2320240121.sapp.pdf]

PNAS

Supporting Information for

G-quadruplex DNA structure is a positive regulator of *MYC* transcription

Isabel Esain-Garcia[1,2,4], Angie Kirchner[1,2,4], Larry Melidis[1,2], Rafael de Cesaris Araujo Tavares[1], Somdutta Dhir[1,2], Angela Simeone[1,2], Zutao Yu[2], Sarah K. Madden[2], Regina Hermann[2], David Tannahill[1], Shankar Balasubramanian[1,2,3*]

[1]Cancer Research UK Cambridge Institute, University of Cambridge, Cambridge CB2 0RE, UK

[2]Yusuf Hamied Department of Chemistry, University of Cambridge, Cambridge CB2 1EW, UK

[3]School of Clinical Medicine, University of Cambridge, Cambridge CB2 0SP, UK

[4]These authors contributed equally

*Shankar Balasubramanian

Email: sb10031@cam.ac.uk

This PDF file includes:

Supporting text
Figures S1 to S23
Tables S1 to S15
Additional methods
SI References

Supporting text

We investigated the minimum number of mutations required to abolish G4 formation at the endogenous genetic context of *MYC* in HEK293T cells. It was essential to consider Pu27 (27 bp, five G-runs) within the context of an extended 48 bp region (i.e. MYC WT, eight G-runs), as there are flanking G-runs that can contribute to G4 folding when central G-runs are mutated (1-3).

Using circular dichroism (CD) spectroscopy, we investigated oligonucleotides (fig. S1A, table S8) consisting of single point mutations within the 27 bp or 48 bp context. Mutating a single G at a time in each of the eight G-runs, resulted in CD spectra with maxima at ~260 nm and minima at ~240 nm, characteristic of G4 structure formation (Permutations 1-13) (4, 5). We then added additional mutations to each G-run starting from the central ones (Permutations 14-20) to establish the threshold of mutations that abrogate G4 structure formation. We found that mutations in each of the eight G-runs within the 48 bp context were needed to completely abolish G4 formation as judged by CD (fig. S1B, permutation 19). We designated permutation 19 as minimally mutated MYC (MUT MIN). Mutations to each of the five central G-runs within the Pu27 core (permutation 18) within the 48 bp context were designated MUT CORE and found to retain canonical G4 spectrum features indicating residual G4 forming potential (fig. S1B). We further explored G4 folding under 10 mM K^+ or Li^+ conditions. G4 oligonucleotides generally show a higher molar ellipticity in K^+ compared to Li^+ (6). We observed no difference in K^+ over Li^+ preference between MYC MUT and MUT MIN suggesting lack of G4 folding. However, MYC WT and MUT CORE showed a preference for K^+ conditions, indicative of G4 folding (fig. S1B).

To confirm that the flanking regions in the MUT CORE oligonucleotide were contributing towards G4 formation, we performed CD in a short 27 bp version of MUT CORE at 10 mM and 100 mM K^+ and observed a profile characteristic of a non-G4 structure (fig. S1C). Overall, this shows the involvement of the three flanking G-runs towards G4 folding in vitro. To measure structural transitions, we deployed UV thermal melting spectroscopy (7). As the 48 bp *MYC* G4 sequence construct had not been previously characterised, we performed experiments at near-physiological conditions (100 mM K^+) and titrated the K^+ concentration (10, 20, 50, 100 mM) to determine that 10 mM K^+ was optimal to capture structural transitions for our constructs in thermal melting measurements (fig. S2A). We measured the UV spectra for MYC WT, MYC MUT, MUT MIN and MUT CORE oligonucleotides at 20°C and 90°C at 10 mM and 100 mM K^+. The thermal difference giving the greatest fold-change between folded and unfolded states was calculated to be ~300 nm (fig. S2B, fig. S2C). Thermal melting measurements were thus taken at this wavelength. MYC MUT and MUT MIN did not display a melting transition (fig. S2D). MUT CORE showed a clear structural transition at ~45°C in 100 mM K^+, whereas MYC WT displayed a transition consistent with greater structural stability (100 mM K^+ at ~60°C) (fig. S2D).

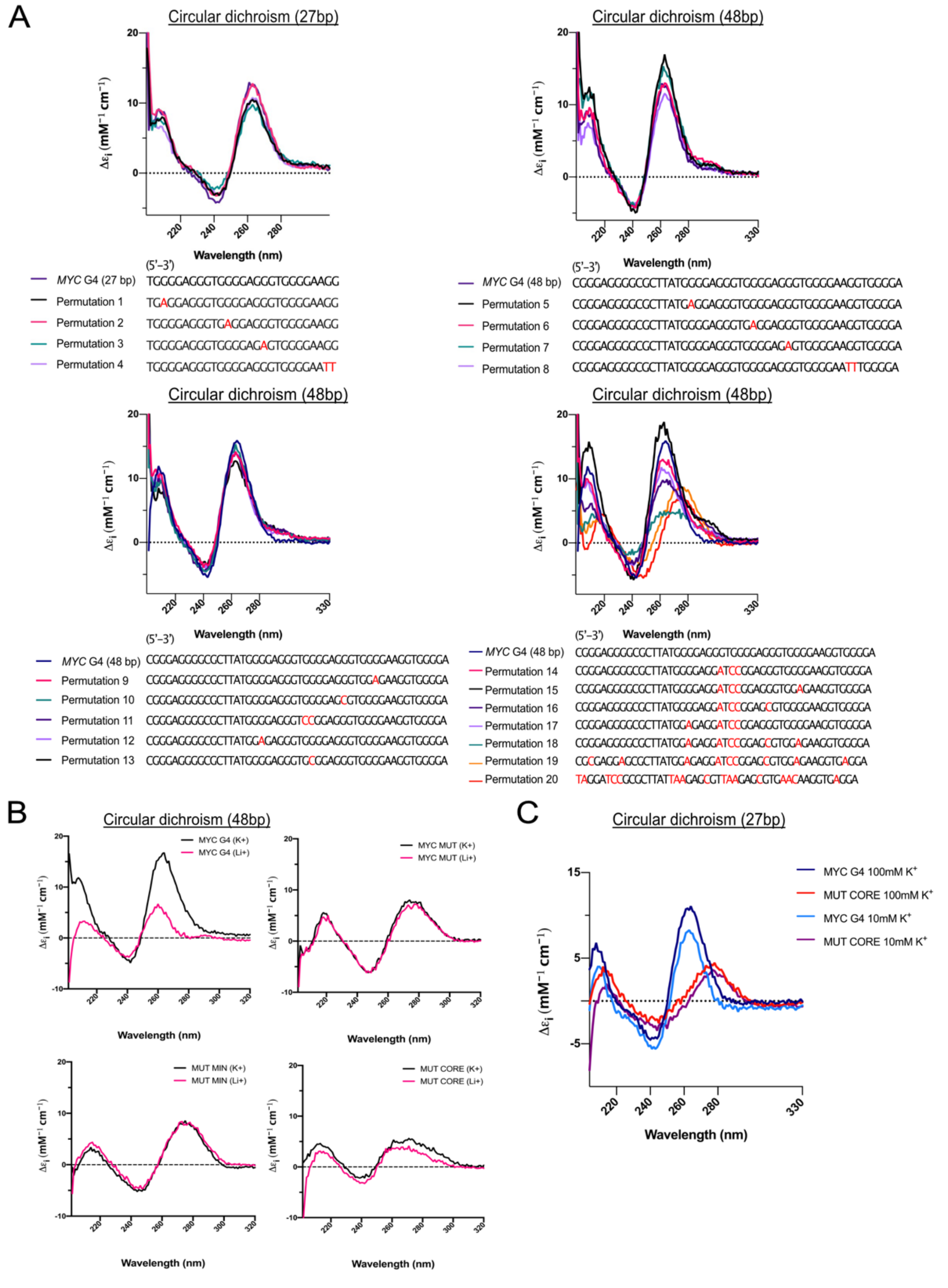

Fig. S1. Circular dichroism *MYC* G4 guanine-contribution mutational study

(*A*) Circular dichroism (CD) for oligonucleotides with different mutations to identify G contributions to G4 folding within the *MYC* G4 27 bp and 48 bp sequence context. A minimum of nine mutations are required for G4 spectral signature loss (Permutation 19). Six G mutations at the core Pu27 sequence retain some G4 forming potential for the 48 bp sequence (Permutation 18). (*B*) Cation-dependency study to interrogate G4 forming in vitro. *MYC* G4 shows a clear

increase in molar ellipticity in the presence of K^+ compared to Li^+. MYC MUT and MUT MIN show no differences between K^+ and Li^+ conditions. MUT CORE shows a degree of K^+ preference. (*C*) CD spectra of MYC G4 and MUT CORE short oligonucleotides (27 bp) in the presence of 10 mM and 100 mM K^+. The G4 spectral signature is lost for the MUT CORE at both K^+ concentrations, and was not lost in the 48 bp context. This result suggests the flanking regions contribute to G4 formation. All measurements were taken in 20 mM lithium cacodylate buffer as previously described (7).

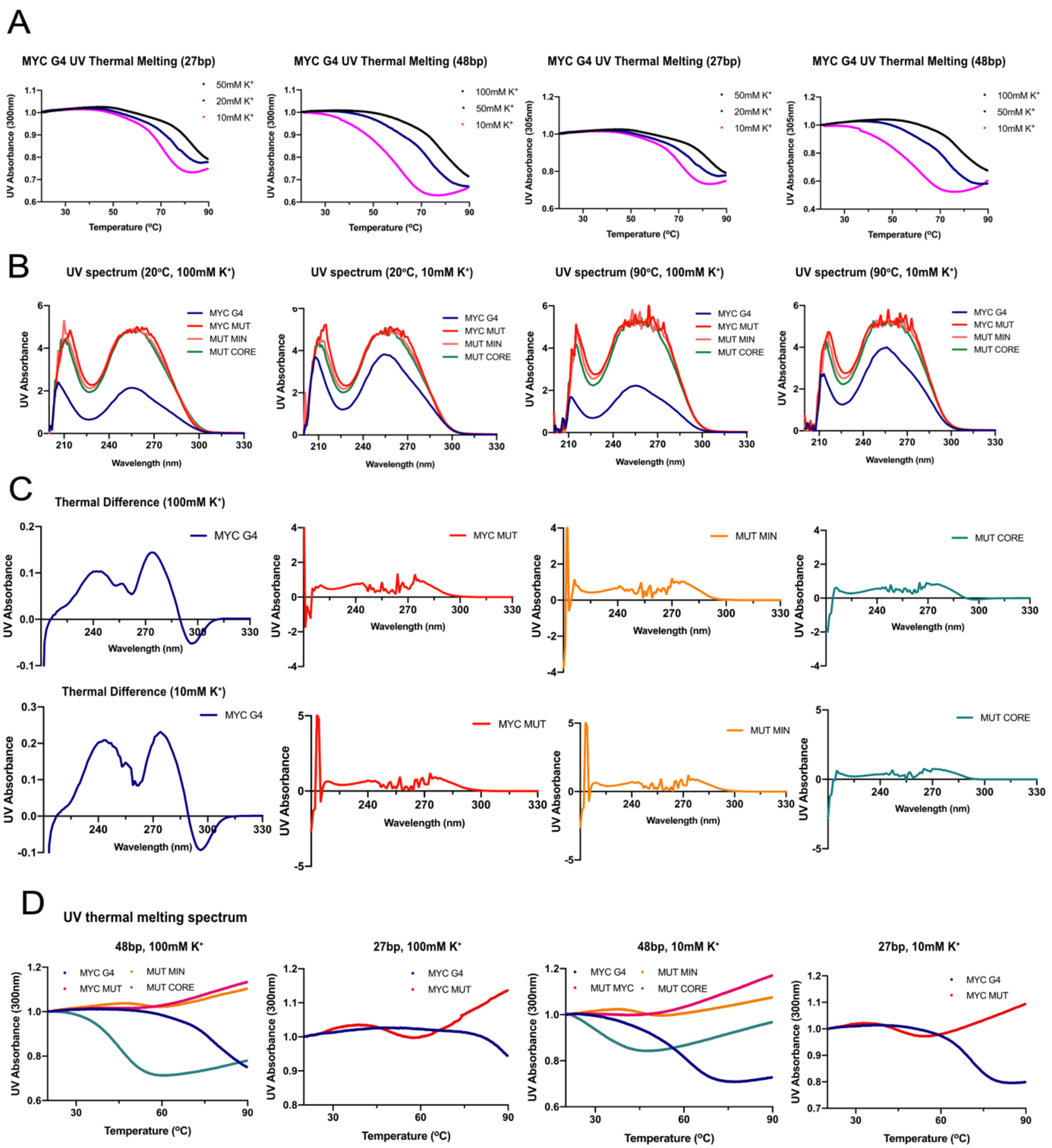

Fig. S2. UV biophysical characterization of the G4 structural perturbations
(*A*) UV thermal melting measurements for the short (27 bp) and long (48 bp) *MYC* G4 sequence context (27 bp, 48 bp) at different K^+ concentrations. Measurements were taken at 300 and 305 nm. (*B*) UV spectrum at 20°C and 90°C for *MYC* G4 (WT), MYC MUT, MUT MIN, MUT CORE at 10 mM and 100 mM K^+. (*C*) Calculated thermal difference spectra for each oligonucleotide at 10 mM and 100 mM K^+. *MYC* G4 shows a minimum at ~300 nm. (*D*) UV thermal melting measurements for the short and long oligonucleotides at 10 mM and 100 mM K^+. 48 bp MUT CORE shows melting at ~45 °C and 48 bp *MYC* G4 at ~69°C. No melting is observed with MYC MUT or MUT MIN. All measurements were taken in 20 mM lithium cacodylate buffer as previously described (7).

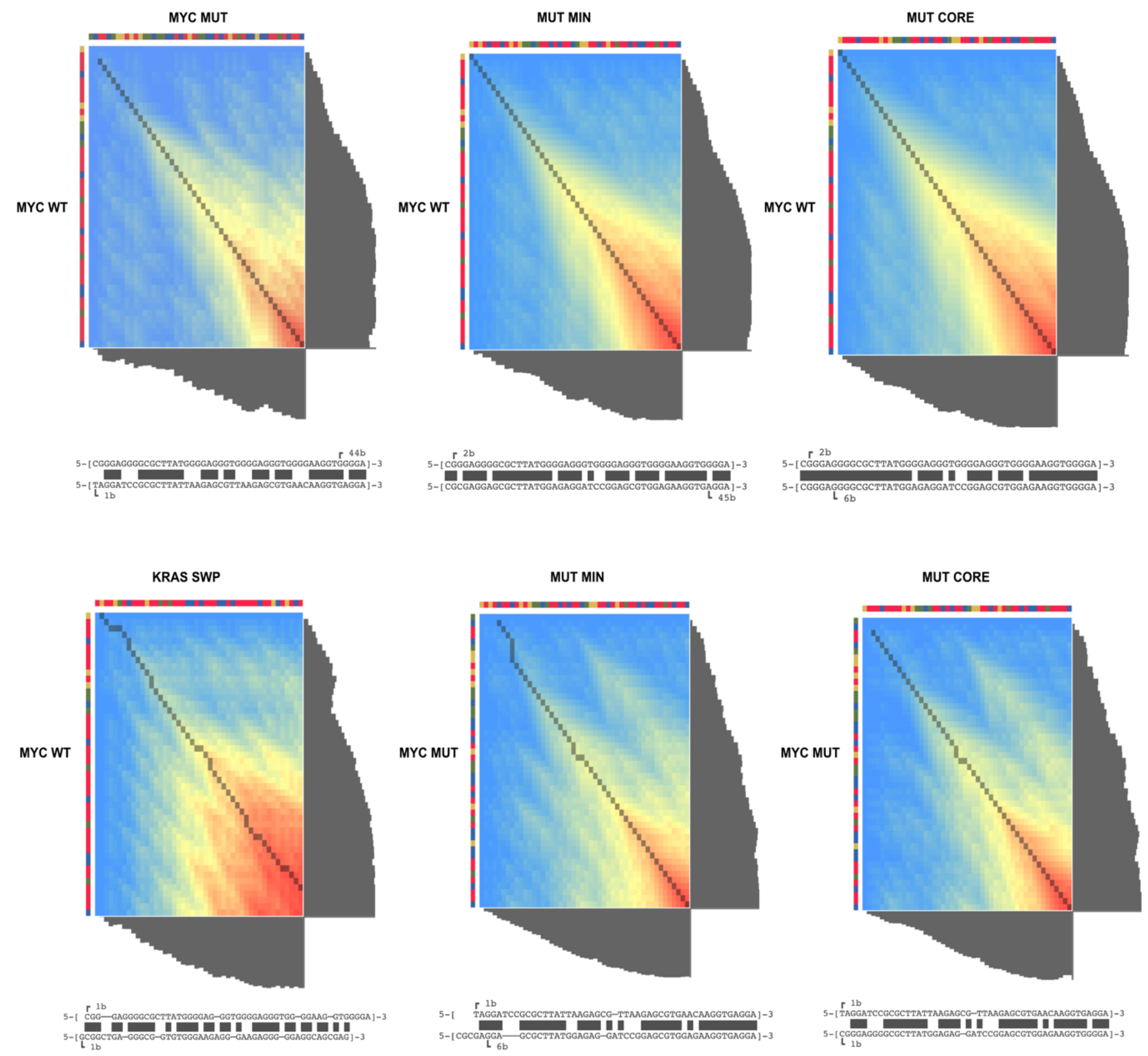

Fig. S3. Assessment of the degree of similarity between DNA sequences
Distance matrixes showing how dissimilar two sequences are when imposing numerical values of matches as penalties for gaps and mismatches based on Needleman-Wunsch algorithm (match = +10, mismatch = -5, gap = -7). This shows that MYC WT and KRAS SWP are dissimilar (NW score = -16) while MYC WT and MYC mutants are similar (NW score = 9).

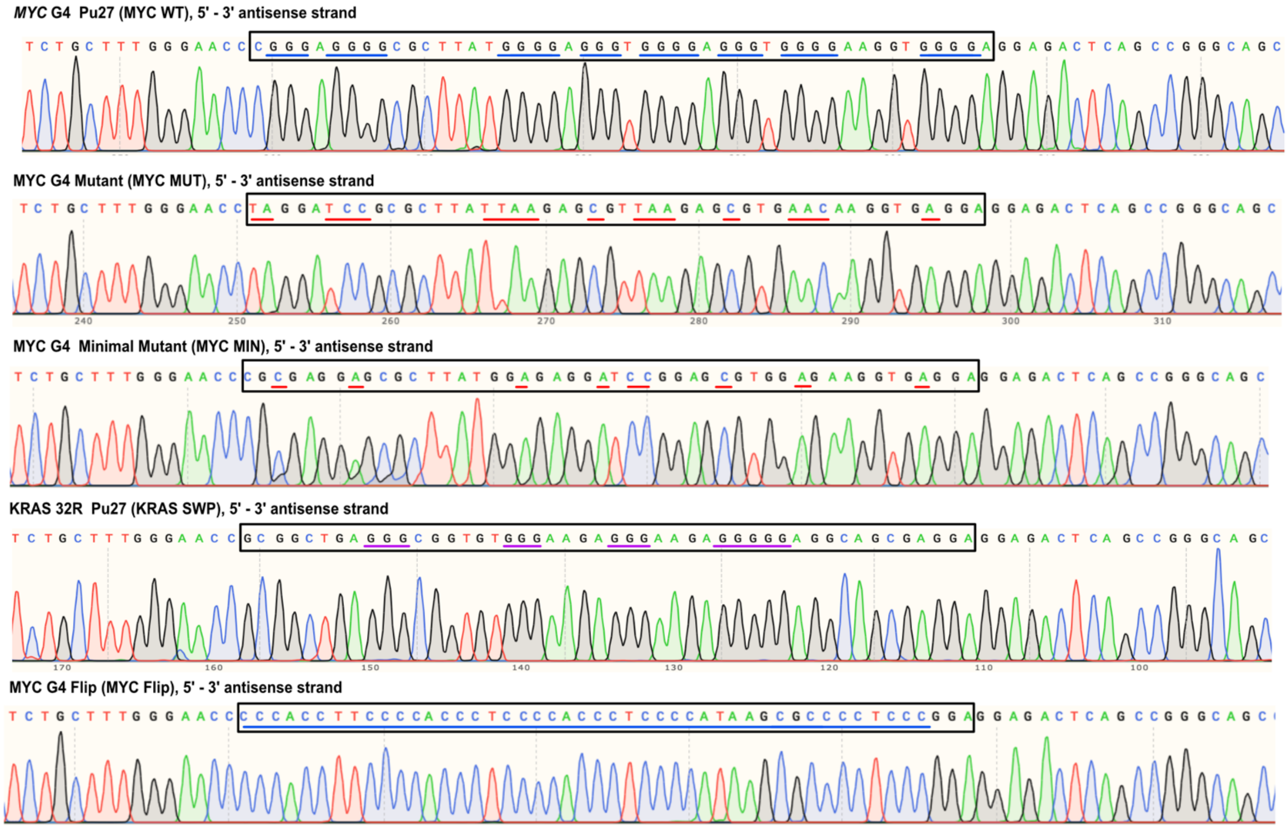

Fig. S4. Genotyping of the generated cell lines for the study
Sanger sequencing chromatograms for an amplicon spanning the edited region (black boxes) and flanking sequences in wild type and edited cell lines. The chromatogram confirms homozygosity of the targeted region in the *MYC* locus for both wild type and edited HEK293T cell lines. Guanine runs (GGG/GGGG) for the wild type *MYC* G4 are underlined in blue (top chromatogram). Point mutations in MYC MUT cells underlined in red. Guanine runs (GGG/GGGGG) for the KRAS SWP are underlined in purple. Sequence for the MYC Flip cell line is highlighted in light blue (bottom track). Guanine (G), Cytosine (C), Thymine (T) and Adenine (A) bases are shown in black, blue, red and green respectively.

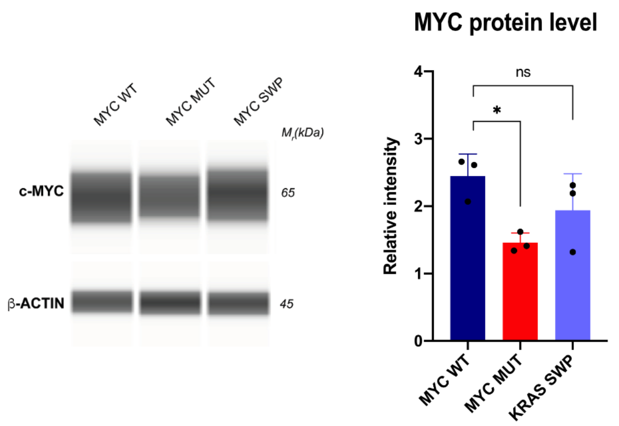

Fig. S5 Western blot analysis of MYC protein levels

Western blot showing a reduction in MYC protein level in MYC MUT cells compared to MYC WT and KRAS SWP (*Left*). The drop in MYC protein intensity (~60% of WT, *: $p \leq 0.05$, ns: not significant, n = 3, *Right*) was estimated as the area-under-the-curve relative to β-ACTIN and P-values were calculated using the Wilcoxon rank-sum exact test.

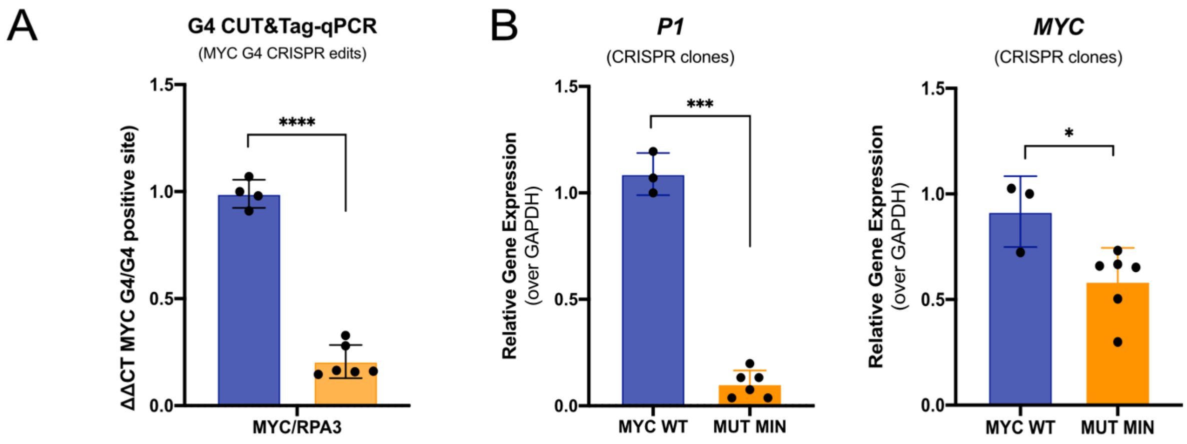

Fig. S6 Characterization of MUT MIN cells

(*A*) MUT MIN cells. G4 CUT&Tag-qPCR showing a decrease of ~80% in signal (P-value < 0.0001) between MYC WT (blue) and MUT MIN (orange), when normalized against the G4-positive site *RPA3*. (*B*) RT-qPCR for P1 and overall *MYC* expression showing significant decrease of ~90% (P-value < 0.0001) and ~41% (P-value = 0.0014), respectively.

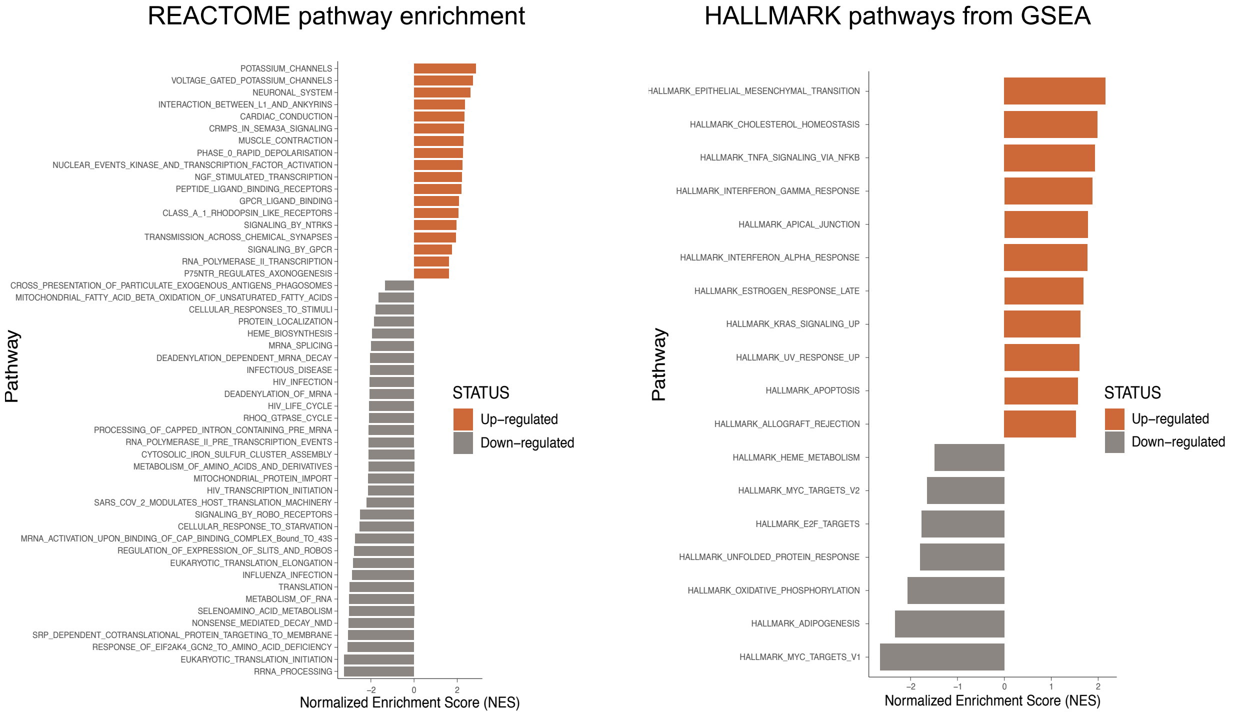

Fig. S7. Ontology analysis for the G4 edited generated cell lines

Pathway enrichment analysis illustrating upregulated and downregulated pathways in the absence of the *MYC* promoter G4 (MYC MUT). Upregulated pathways include signaling pathways and pro-apoptotic programs. Downregulated pathways include are MYC targets, mRNA splicing and translation. The score is calculated using gene set enrichment analysis (GSEA) as normalized enrichment score (NES).

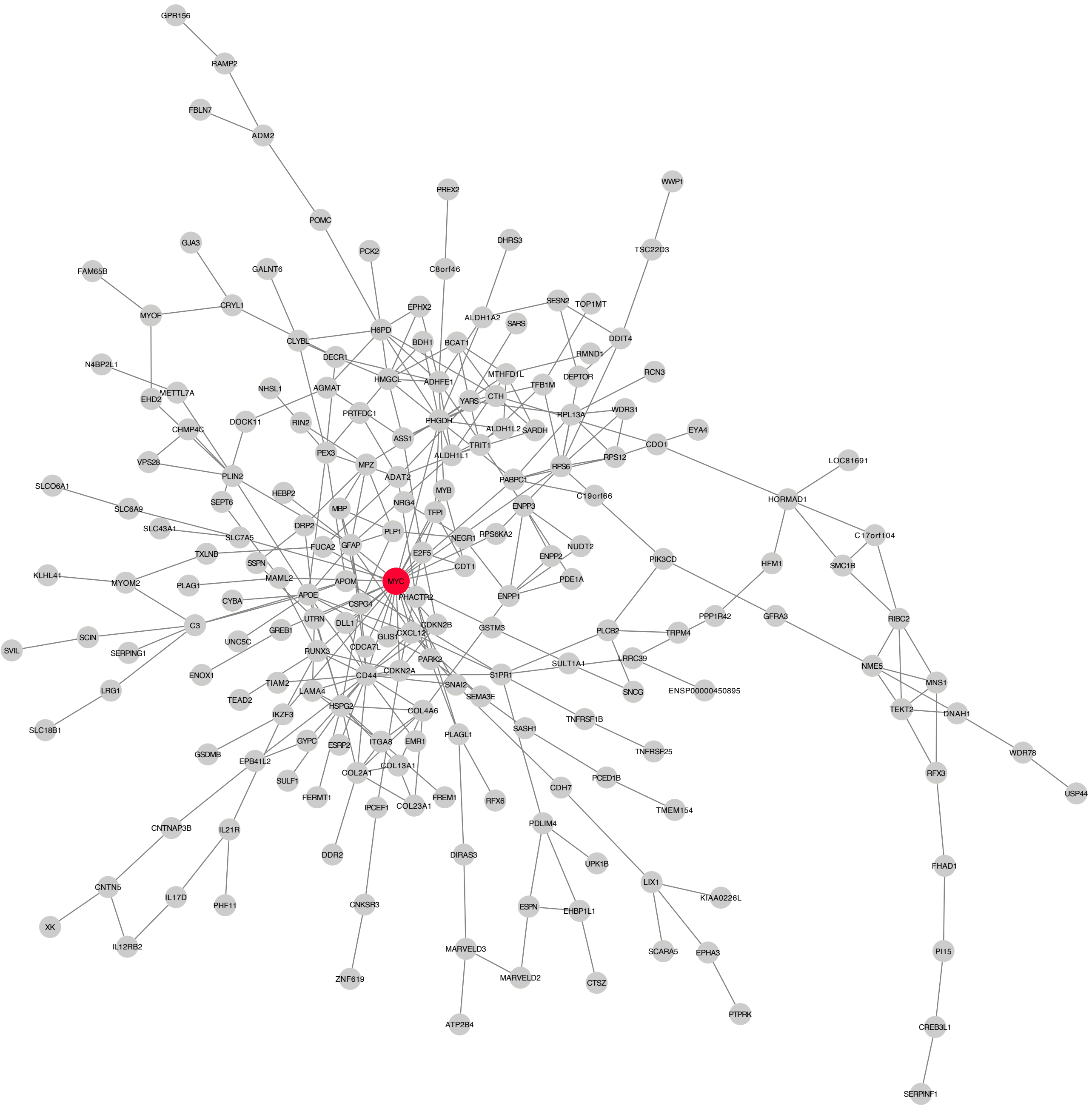

Fig. S8. Network diagram for MYC downregulated hubs

Protein-protein interaction (PPI) network diagram between all downregulated genes in the MYC MUT cells (n=459, qval ≤ 0.05 and FC ≤ 0.5) using Cytoscape. Each node represents a protein and each line refers an interaction.

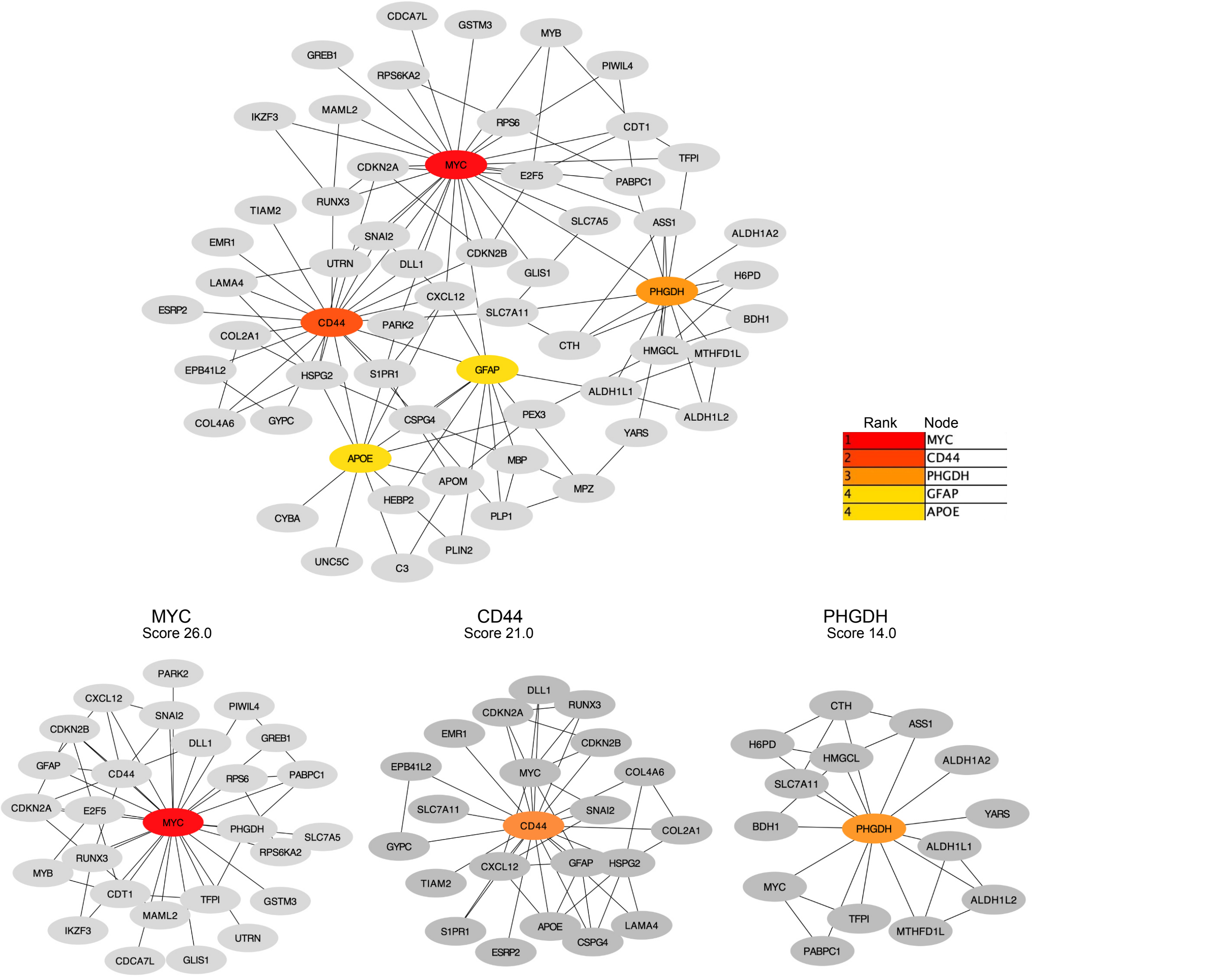

Fig. S9. Network diagram for MYC downregulated hubs

PPI network showing the interaction of top 200 downregulated genes in the MYC MUT cells (qval ≤ 0.05 and FC ≤ 0.5). *MYC* is the most downregulated hub (Score 26.0), followed by *CD44*, *PHGDH*, *GFAP* and *APOE* in the ranking order of statistical significance (qval ≤ 0.05 and FC ≤ 0.5).

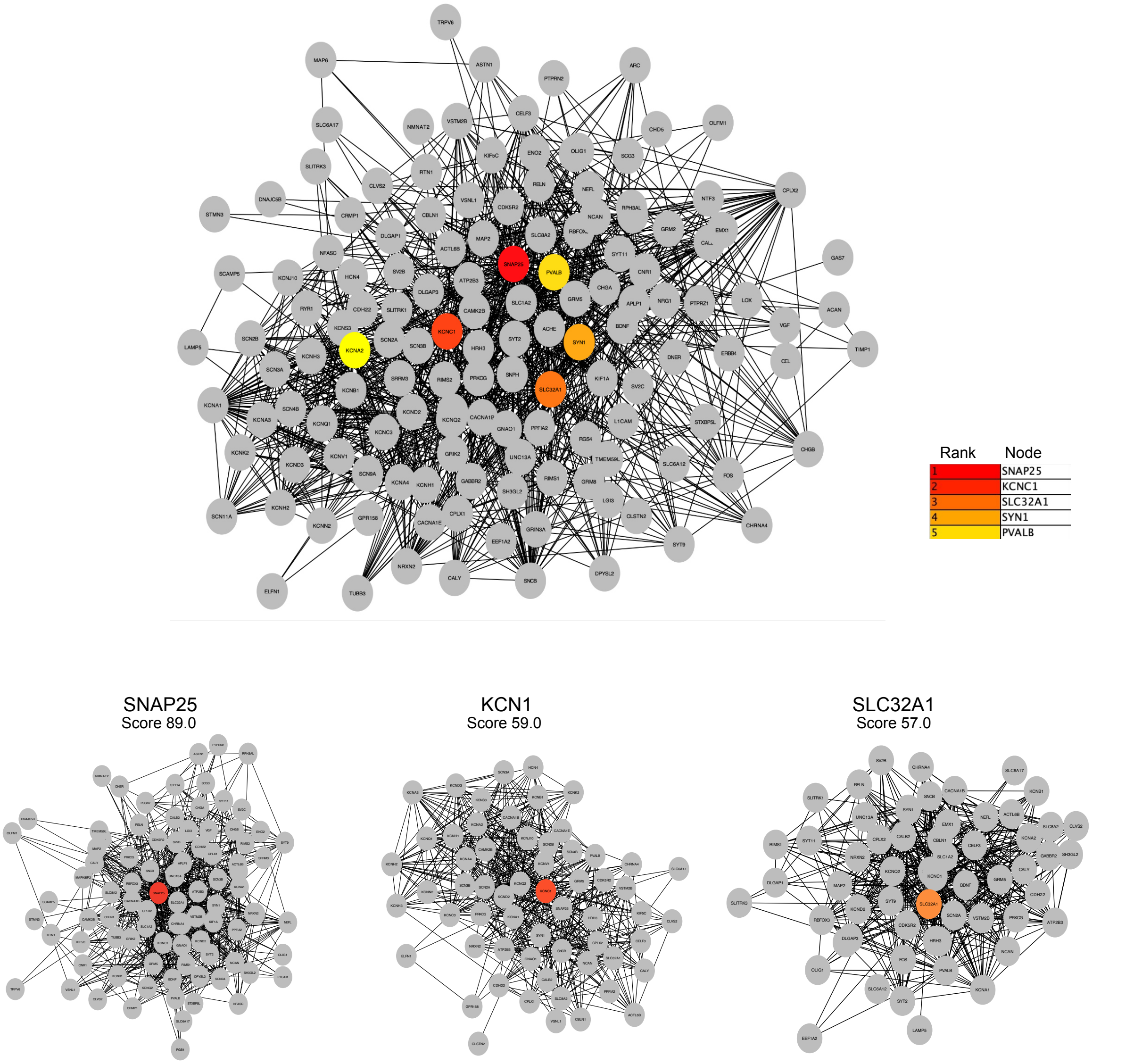

Fig. S10. Network diagram for MYC upregulated hubs

PPI network diagram illustrating the interactome hub of the most upregulated genes (n = 200). These genes are *SNAP25*, *KCN1* and *SLC32A1* in the ranking order of statistical significance (qval ≤ 0.05 and FC ≤ 0.5).

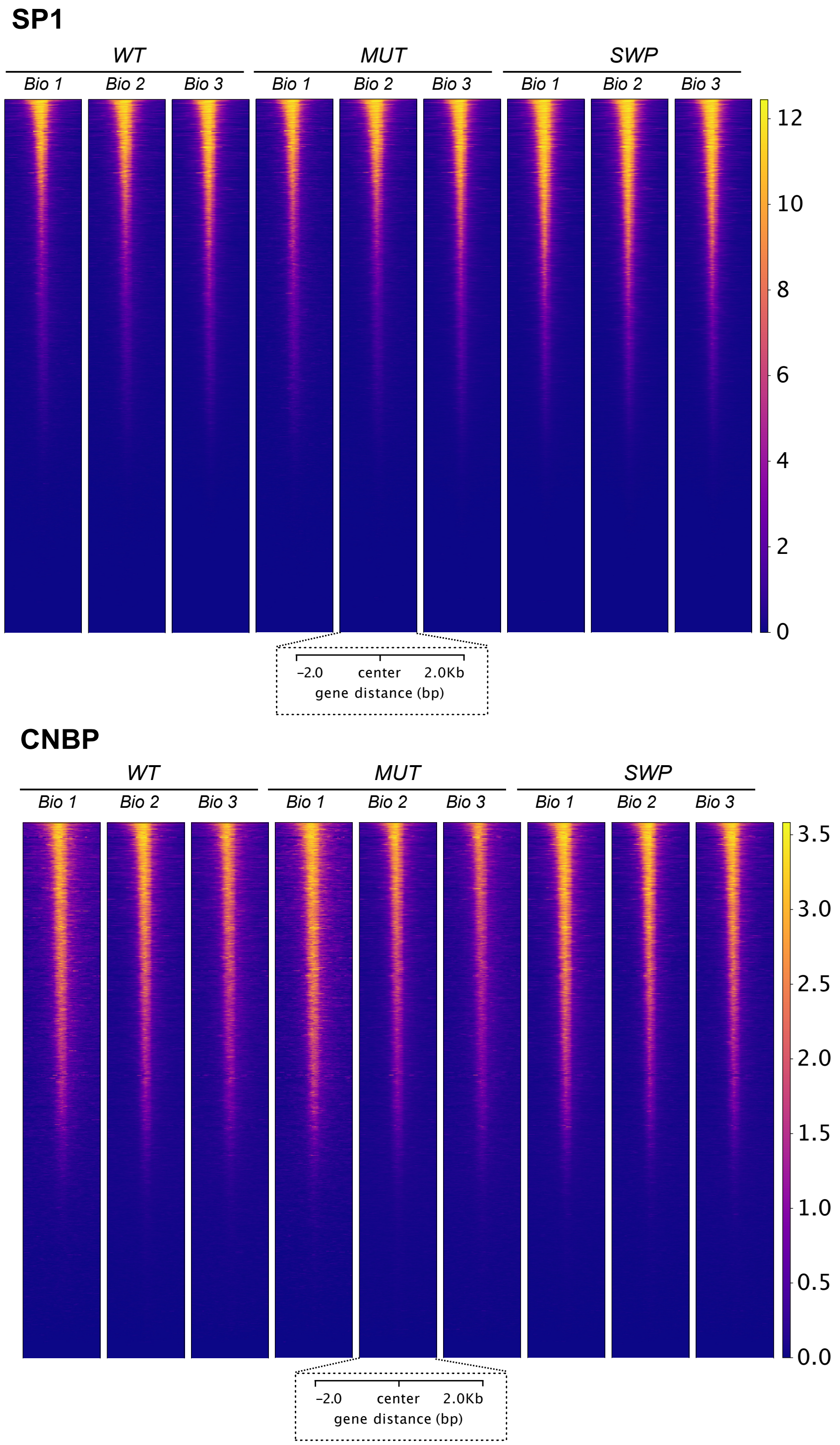

Fig. S11. Heatmaps of the binding of transcription factors SP1 and CNBP in respect to G4 sites
Heatmaps showing genome-wide similarities in G4 profile across MYC WT, MYC MUT and KRAS SWP cells. The tracks show normalized coverage values. Profiles are centered at G4s and cover +/- 2Kb.

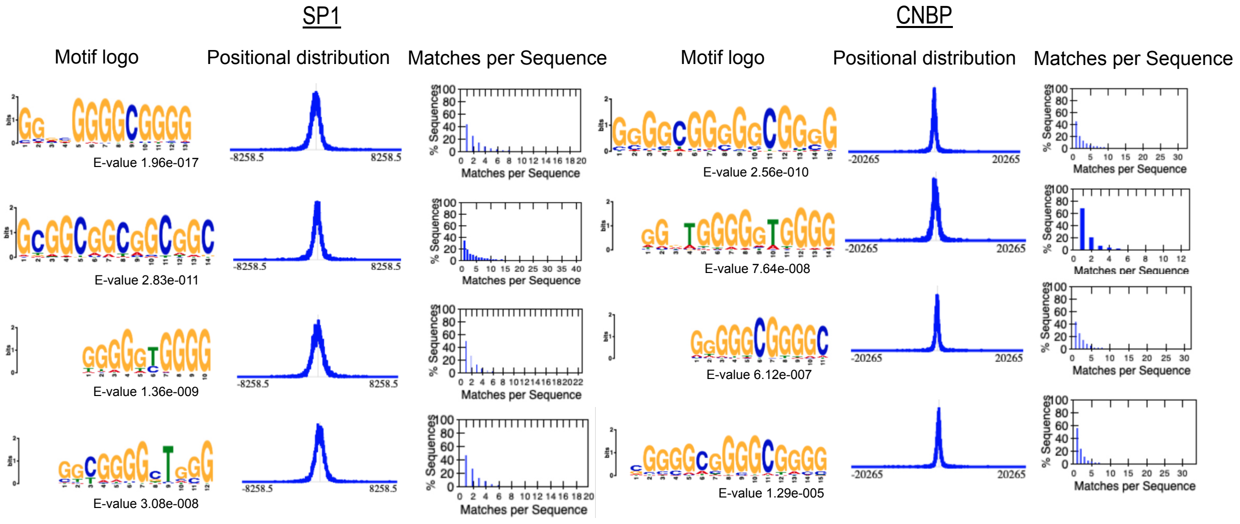

Fig. S12. Motif analysis for G4 binders

Motif analysis derived from the generated CUT&Tag data showing the binding sequences for SP1 and CNBP. The preferential binding motifs include G-rich sequences which can fold into G4s.

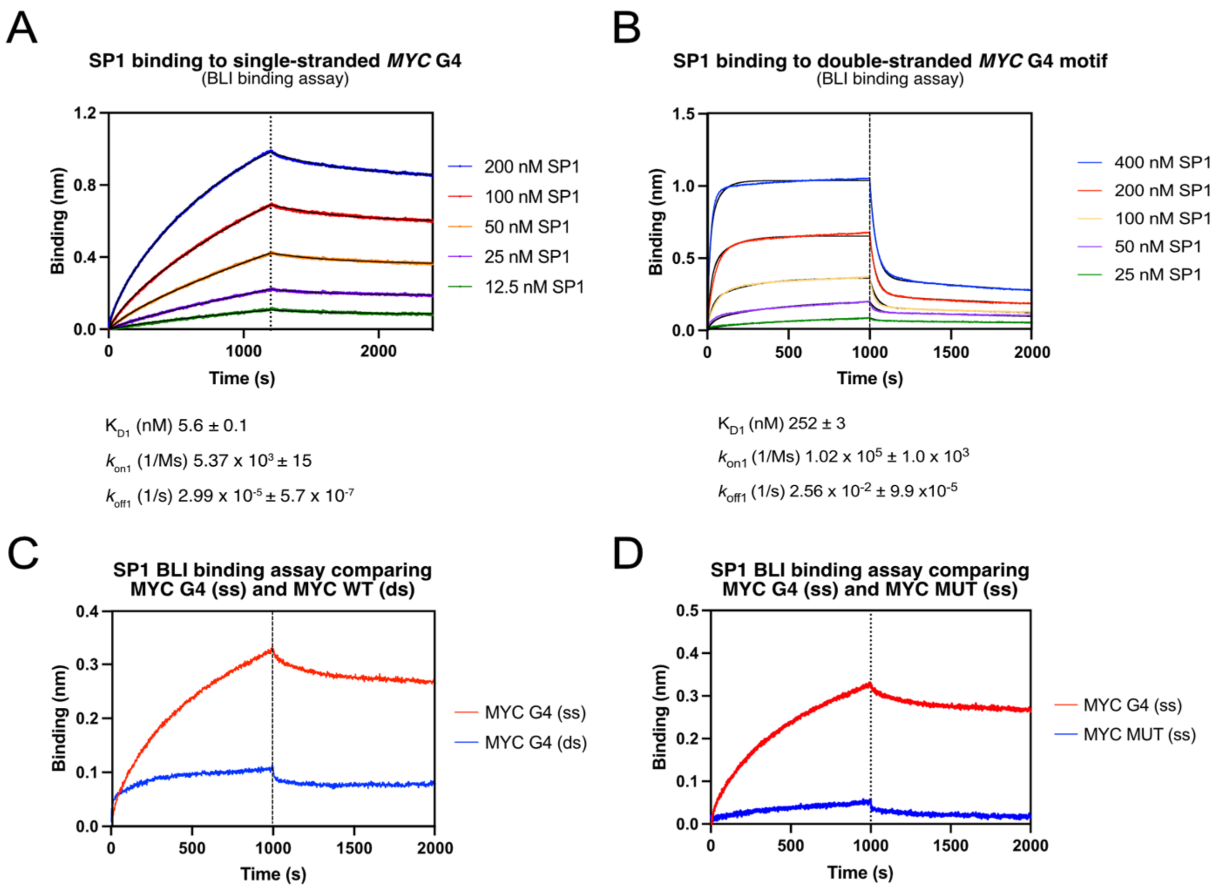

Fig. S13. BLI assays to calculate the binding affinity of SP1 to the double strand (ds) and single strand (ss) *MYC* G4 and *MYC* MUT

Biolayer Interferometry (BLI) analysis confirms the SP1-G4 interaction. (*A*) Binding curves illustrating the association and dissociation of recombinant SP1 protein to the immobilized, biotinylated ssDNA oligonucleotide containing the *MYC* G4. Apparent K_D for the most abundant population is indicated. (*B*) As in (*A*) but for dsDNA oligonucleotide containing the *MYC* G4 motif. (*C*) Binding curve showing the association and dissociation of SP1 to the single-stranded *MYC* G4 and double-stranded *MYC* G4 motif at 100 nM SP1 protein. SP1 preferentially binds to G4-forming ssDNA. (*D*) As in (*C*) but for *MYC* G4 and the *MYC* MUT ssDNA. SP1 preferentially binds to G4-forming ssDNA.

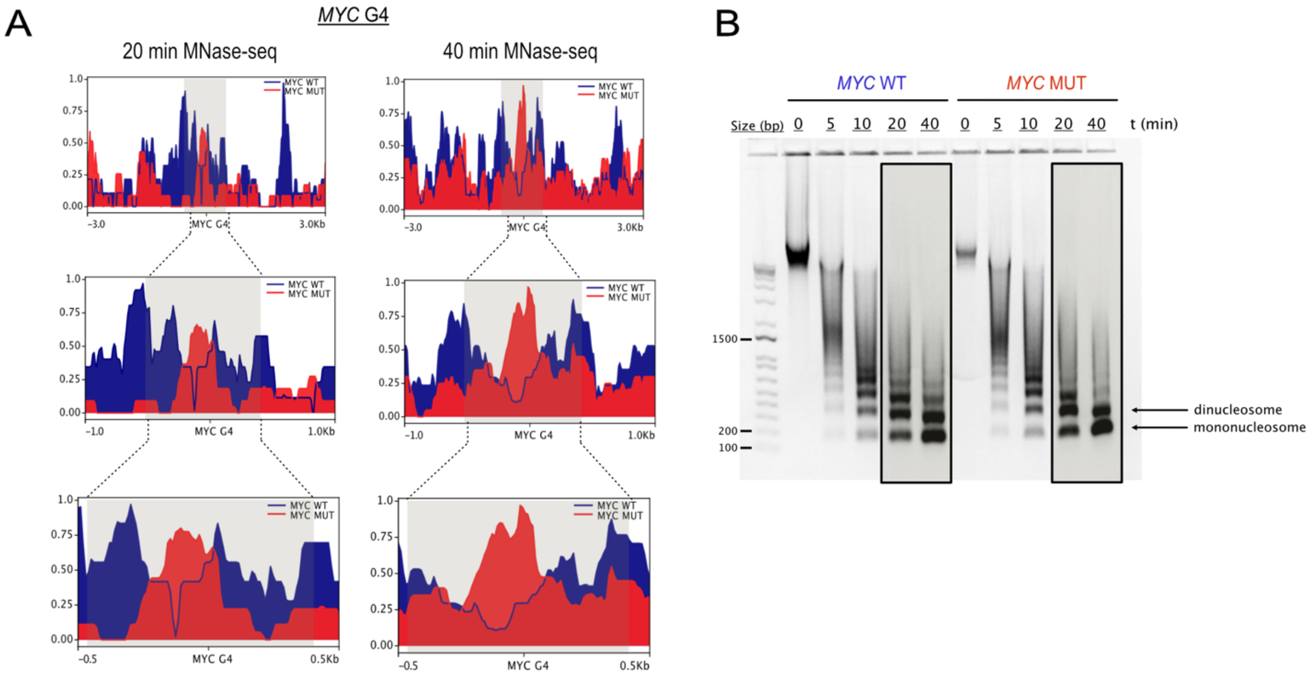

Fig. S14. Nucleosome positioning by G4 structures

(*A*) MNase-seq genome tracks showing nucleosome positioning at the G4 edited site across different MNase digestion time points (20 min, 40 min). (*B*) 2% Agarose gel with the MNase-digested genomes of MYC WT and MYC MUT cells after 5, 10, 20 and 40 min of digestion at 37°C. The bottom bands correspond to the mono- and di-nucleosomal fragments.

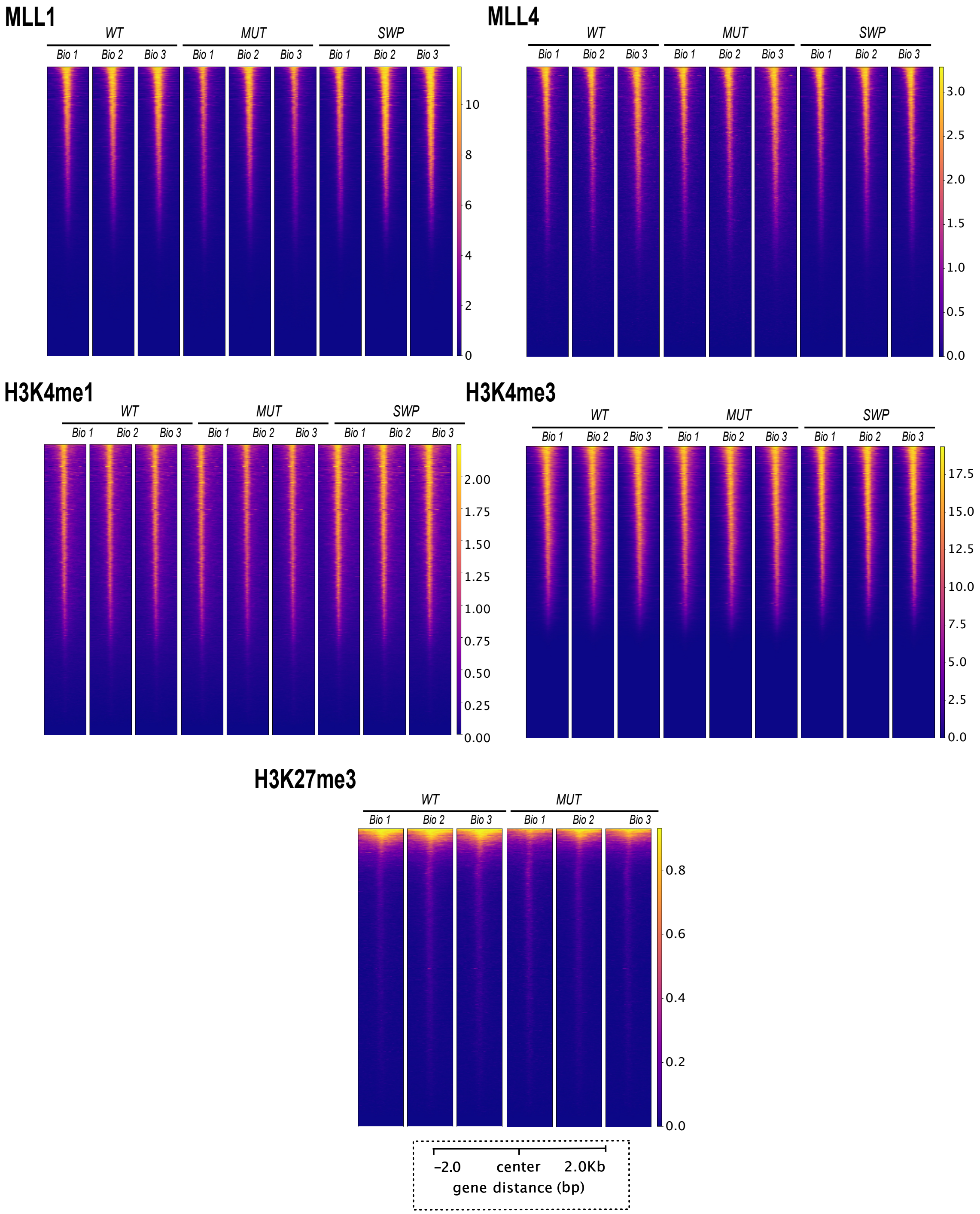

Fig. S15. Heatmaps of the binding of histone modifiers and histone methylation distribution in respect to G4 sites

Heatmaps showing genome-wide binding of histone methyltransferase MLL1 and activating H3K4me1 and H3K4me3 marks to G4 sites. A repressive mark (H3K27me3) shows no G4 overlap. Tracks show normalized coverage values. Profiles are centered at G4s and cover +/- 2Kb.

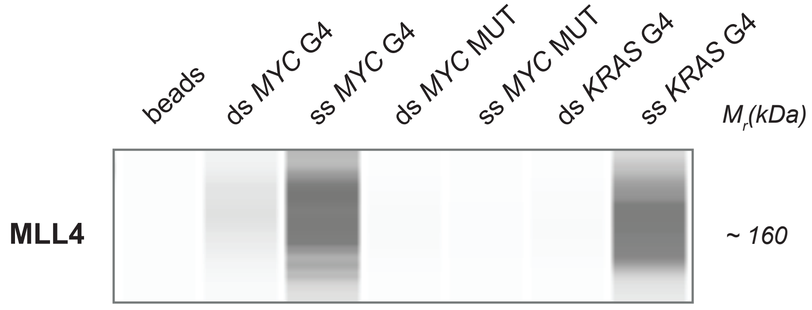

Fig. S16. MLL4 protein affinity enrichment by G4 folded oligonucleotides and controls
Affinity enrichment and western blot analysis for MLL4 protein for double strand (ds) and single strand (ss) *MYC* G4, ss/ds *MYC* MUT, ss/ds and *KRAS* G4.

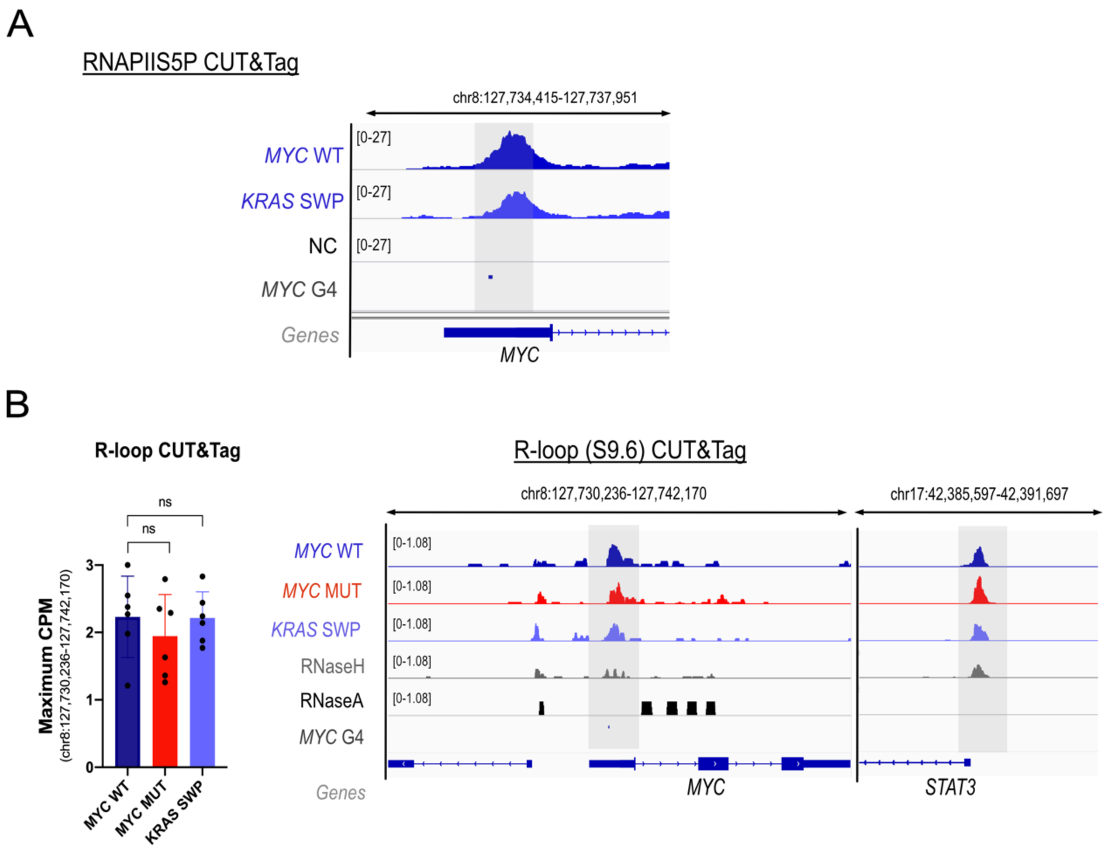

Fig. S17. RNAPIIS5P and R-loop CUT&Tag

(*A*) MYC WT and KRAS SWP genomic binding profiles (IGV tracks) showing RNAPII CUT&Tag for transcriptional initiation (RNAPIIS5P). (*B*) R-loop (S9.6) CUT&Tag sequencing profile (Integrative Genomics Viewer, IGV tracks, *Right*) and quantification showing no statistically significant changes when compared to MYC WT between MYC MUT (P-value = 0.4378) and KRAS SWP (P-value = 0.9603). RNase H and RNase A digestions were included as controls. Bar chart illustrating three biological replicates with two technical replicates each (*Left*).

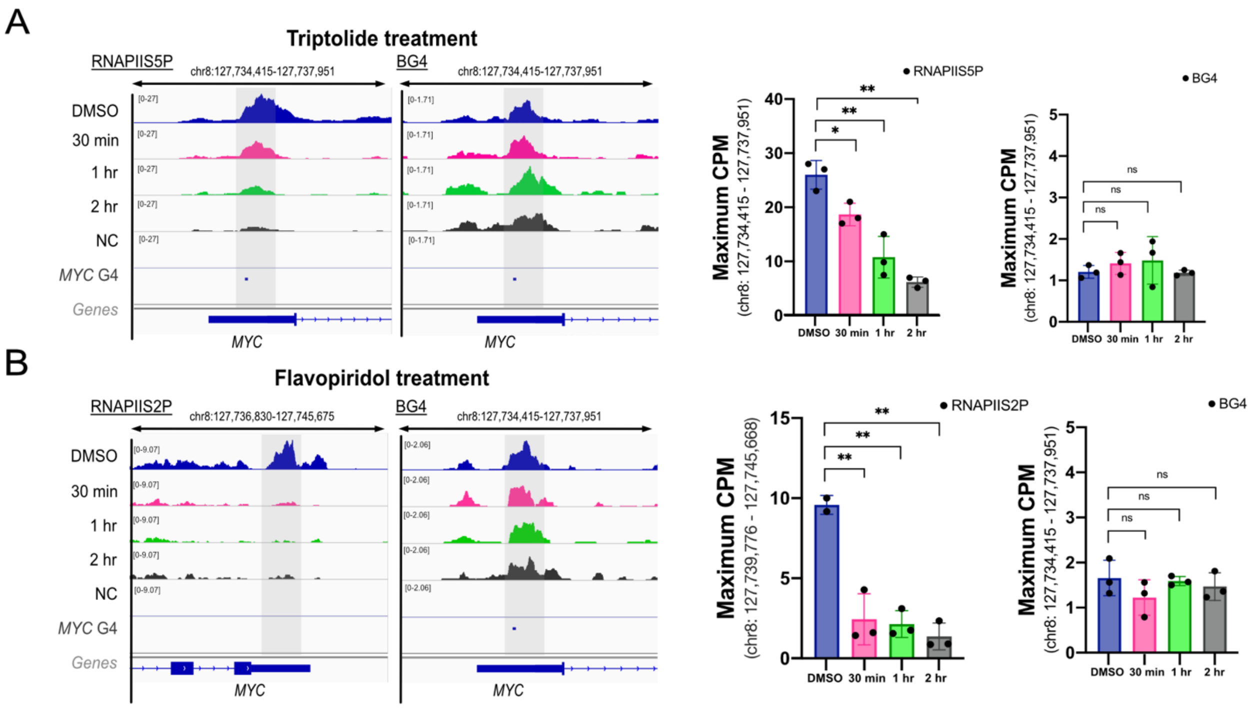

Fig. S18. G4 and RNAPII CUT&Tag upon transcriptional inhibition

(*A*) Sequencing tracks illustrating RNAPIIS5P and BG4 signal upon transcriptional initiation inhibition with triptolide over time (left), and its respective quantification (right). A statistically significant drop in RNAPIIS5P signal at the *MYC* locus over time (30 min 28.20%, P-value = 0.0216; 1 h, 58.56%, P-value = 0.0069 and 2 h, 76.24%, P-value = 0.0027), as well as genome-wide. Conversely, no statistically significant changes to G4 formation were observed for any of the time points (30 min P-value = 0.3312, 1 h P-value = 0.4950, 2 h P-value = 0.8278). (*B*) As in (*A*) but upon transcriptional elongation inhibition with flavopiridol. A statistically significant time-dependent drop in RNAPIIS2P signal (30 min, 74.54%, P-value = 0.0084; 1 h, 77.64%, P-value = 0.0016 and 2 h, 85.71% P-value = 0.0012). No statistically significant changes to G4 formation were observed for any time point (30 min P-value = 0.2492, 1 h P-value = 0.8004, 2 h P-value = 0.5469).

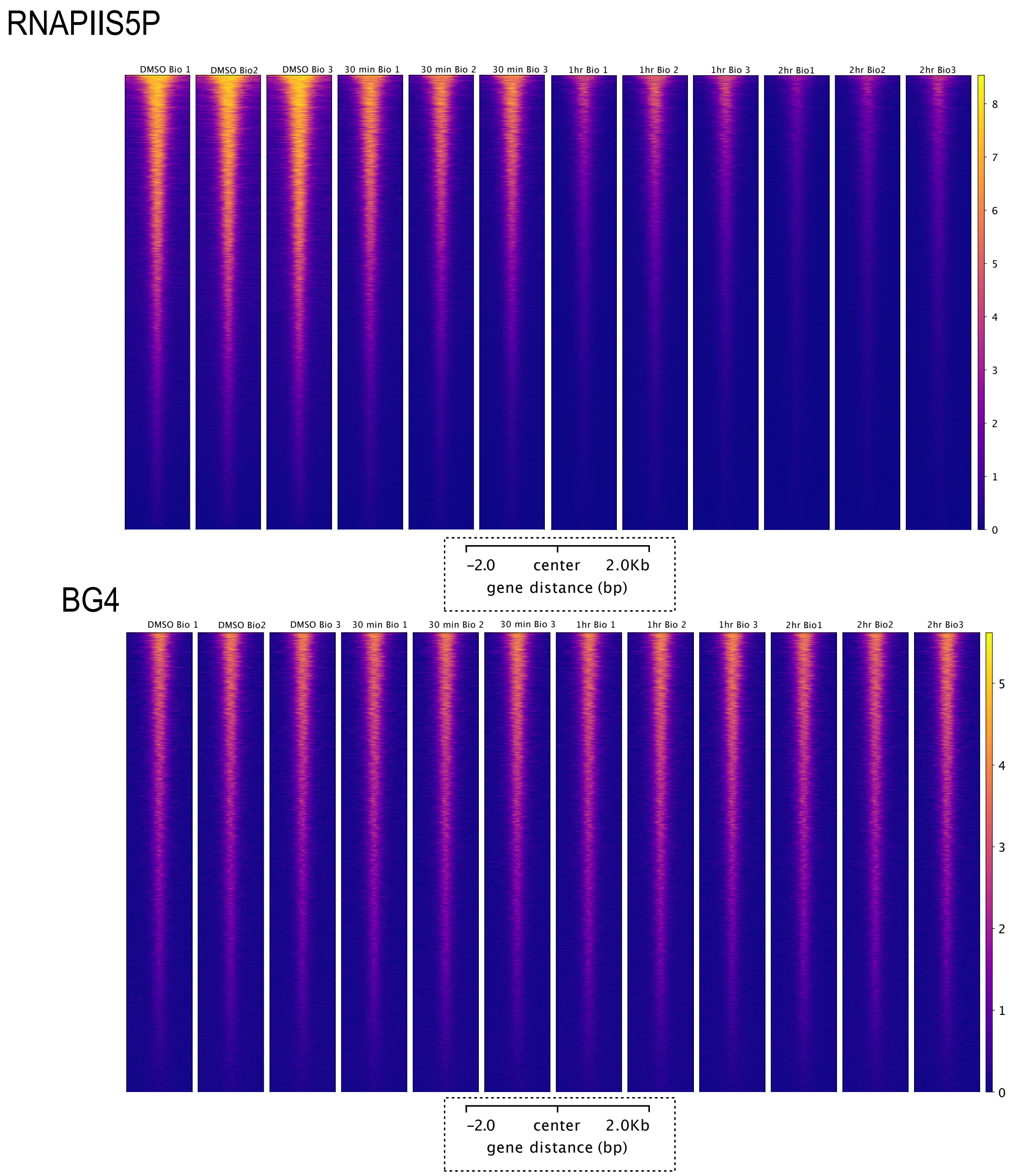

Fig. S19. Heatmaps of the RNAPIIS5P and BG4 signals upon a triptolide treatment

Heat maps showing genome-wide binding of RNAPII and BG4 at transcription start sites (TSS). The tracks show normalized coverage values. RNAPIIS5P signal is lost over time upon treatment. Profiles are centered at G4s and cover +/- 2Kb.

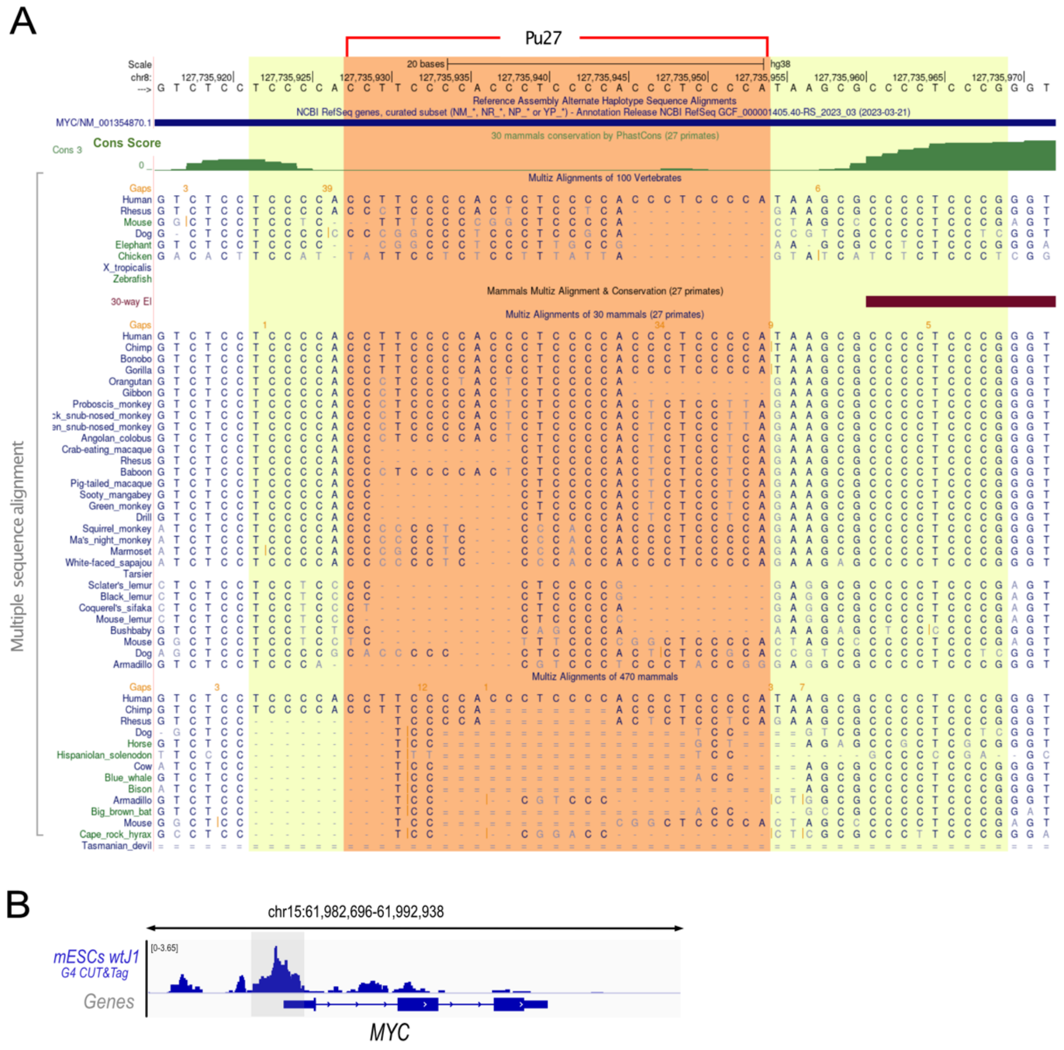

Fig. S20. Sequence conservation analysis on the *MYC* locus

(*A*) Comparison of the *MYC* Pu27 sequence across different species. Higher primates show sequence conservation while other species do not. (*B*) Genomic binding profiles (IGV tracks) showing the BG4 CUT&Tag signal in mouse embryonic stem cells (J1) mapped on the mm10 gene build. The enrichment at the *MYC* locus is indicative of G4 formation.

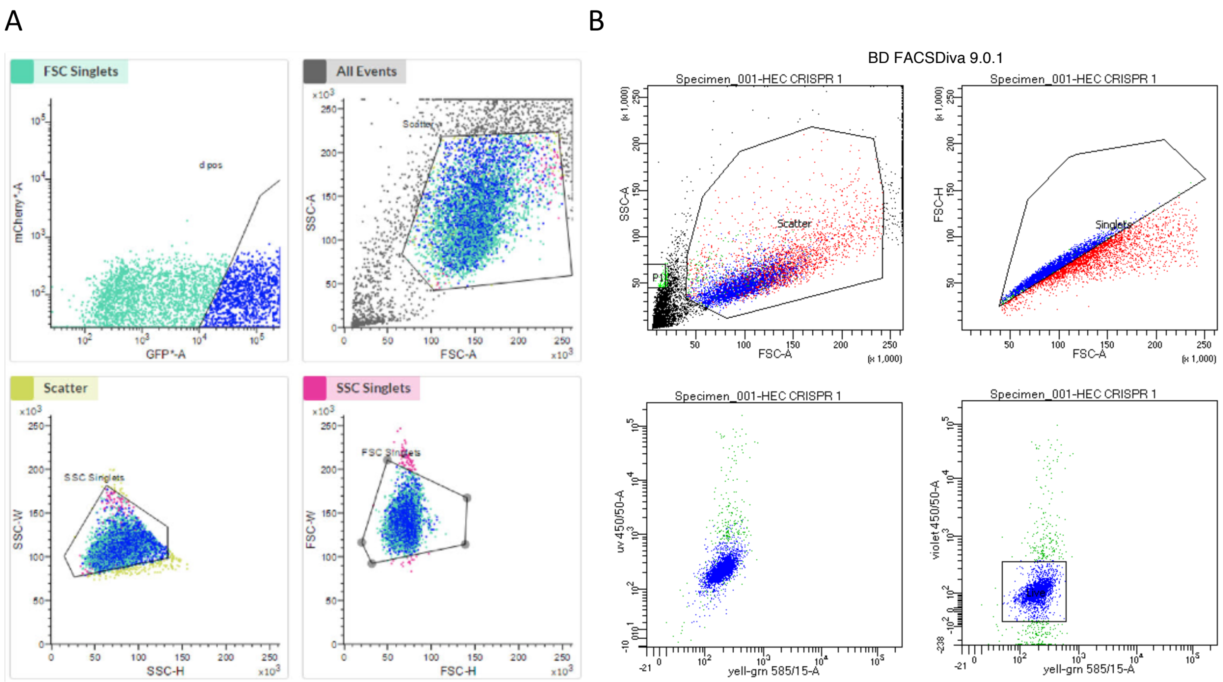

Fig. S21. FACS sorting of single CRISPR clones

(*A*) Transfection-based editing. Plots of gating strategy for sorting GFP-positive transfected cells. FSC Singlets (top left) illustrate the sorted cells. Cells in blue (stringent gating) were collected for CRISPR subcloning. For 6,093 events, parent = 98.56%, FSC-A Median = 150876.58. All events (top right) showing the total population profile (Total = 100%, FSC-A Median = 155482.08). Scatter plot (bottom left) illustrating the singlet population SSC-W/SSC-H (parent = 63.50%, FSC-A Median = 150762.63). SSC singlets (bottom right) showing the singlet population FSC-W/FSC-H (parent = 97.35%, FSC-A Median = 151146.69). (*B*) Electroporation-based editing. The electroporated population was sorted into single cells for CRISPR subcloning.

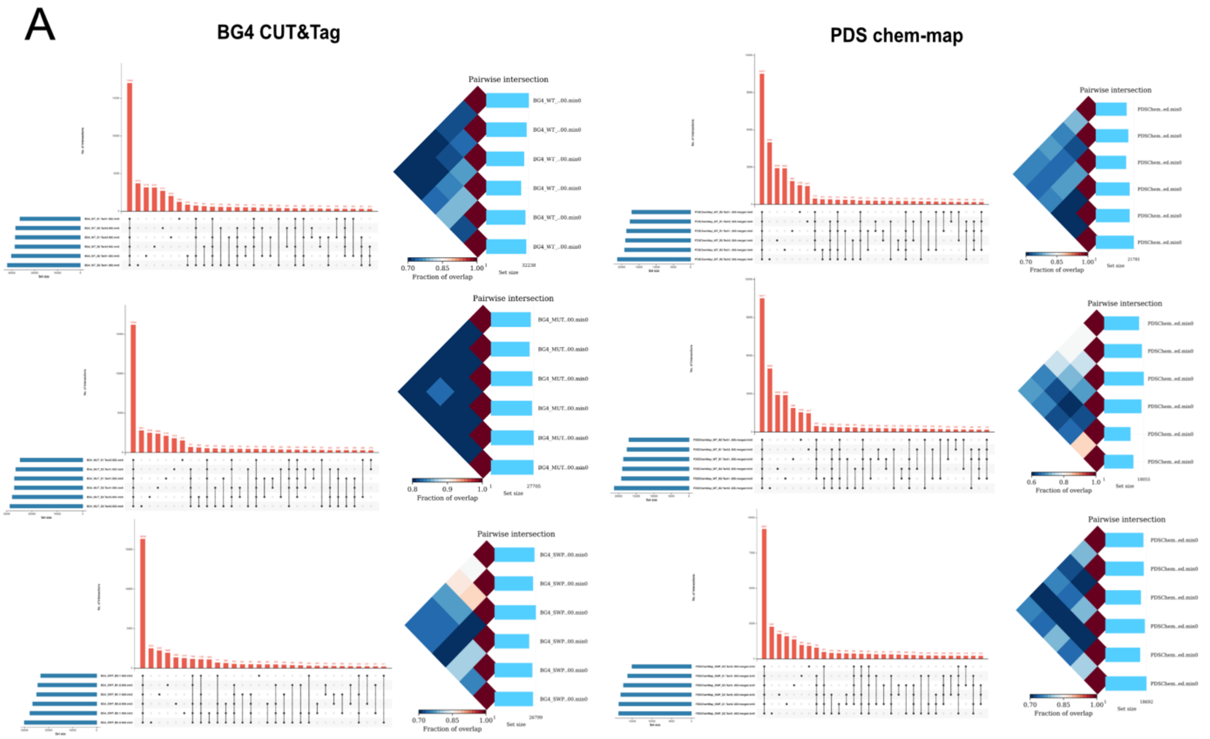

Fig. S22. Peak overlap across biological samples for BG4 CUT&Tag and PDS chem-map
UpSet plots and pairwise diagrams illustrating the overlap across two technical replicates and three biological samples for BG4 CUT&Tag and PDS chem-map. The most abundant subset is the one where the peak is present in six out of the six family replicates, indicating high technical reproducibility across samples.

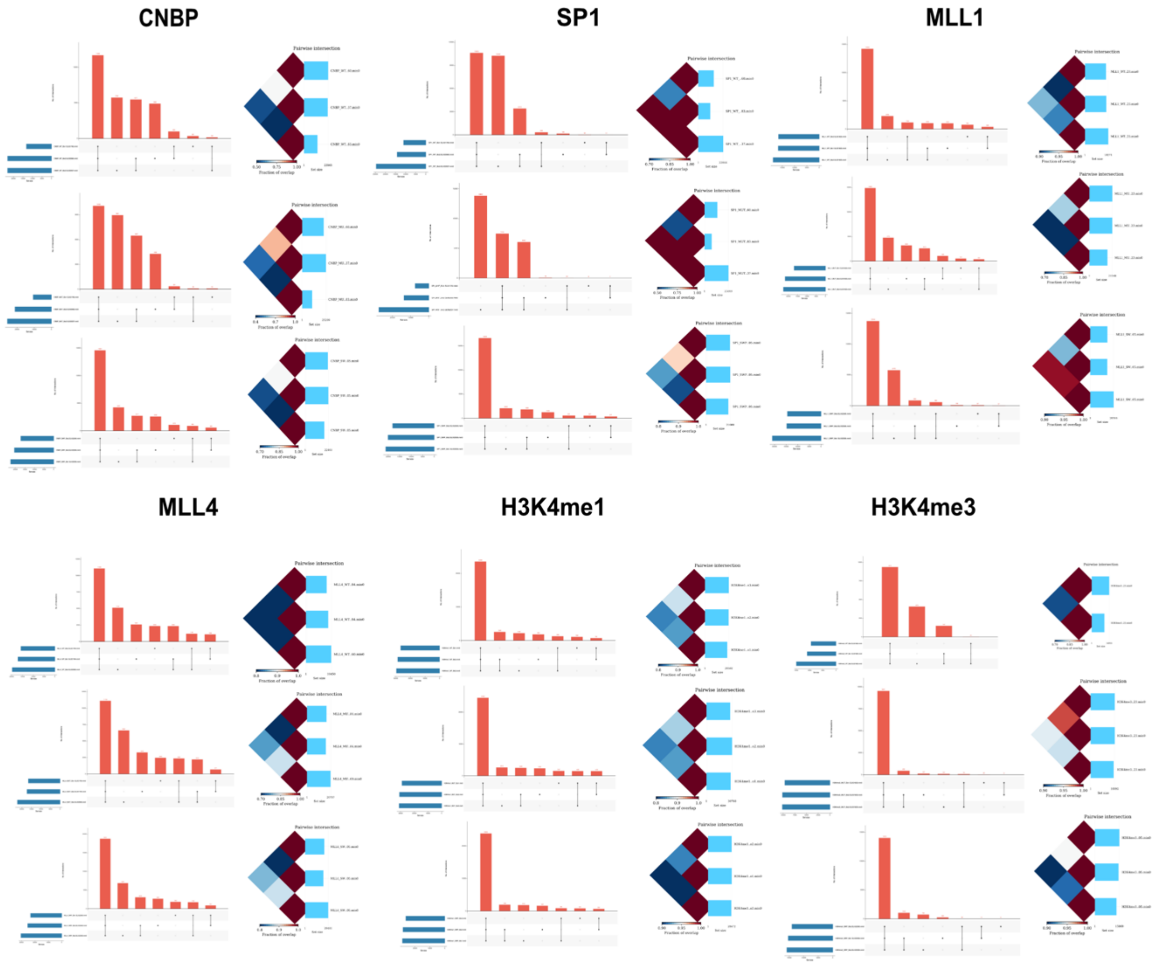

Fig. S23. Peak overlap across biological samples for the MYC interactome

UpSet plots and pairwise diagrams illustrating the overlap across three biological samples for CUT&Tag of CNBP, SP1, MLL1, MLL4, H3K4me1 and H3K4me3. The most abundant subset is the one where the peak is present in three out of the three family replicates, indicating high technical reproducibility across samples.

Table S1. Statistical test on CUT&Tag and Chem-map qPCR at the *MYC* G4 locus

**BG4 CUT&Tag-qPCR**	**P-value**	**Welch-corrected, two-tailed, student t-test**
MYC WT/MYC MUT RPA3	0.033(**)	t=16.32, df=2.060
MYC WT/KRAS SWP RPA3	0.0199(*)	t=4.858, df=2.777
MYC WT/MYC MUT MAZ	0.0009(***)	t=25.95, df=2.192
MYC WT/KRAS SWP MAZ	0.0073(**)	t=7.024, df=2.816
MYC WT/MYC MUT RBBP4	0.005 (***)	t=16.75, df=2.918
MYC WT/KRAS SWP RBBP4	0.1295(ns)	t=1.905, df=4.000
MYC WT/KRAS SWP RPA3	0.0010(**)	t=7.246, df=4.678
MYC WT/MUT MIN RPA3	<0.0001(****)	t=17.20, df=7.330
**PDS ChemMap-qPCR**		
MYC WT/MYC MUT RPA3	0.0007(***)	t=26.25, df=2.297
MYC WT/KRAS SWP RPA3	0.0058(**)	t=5.957, df=3.548
MYC WT/MYC MUT MAZ	<0.0001(****)	t=65.66, df=2.724
MYC WT/KRAS SWP MAZ	0.0119(*)	t=6.461, df=2.569
MYC WT/MYC MUT RBBP4	<0.0001(****)	t=57.92, df=2.772
MYC WT/KRAS SWP RBBP4	0.1391(ns)	t=2.382, df=2.016
**SG4 CUT&Tag-qPCR**		
MYC WT/MYC MUT RPA3	<0.0001(****)	t=18.69, df=3.972
MYC WT/MYC SWP RPA3	0.1324 (ns)	t=2.118, df=2.755
MYC WT/MYC MUT MAZ	0.0074(**)	t=10.60, df=2.103
MYC WT/MYC SWP MAZ	0.0241(*)	t=4.026, df=3.217
MYC WT/MYC MUT RBBP4	0.0005(***)	t=20.28, df=2.705
MYC WT/MYC SWP RBBP4	0.4415 (ns)	t=0.8805, df=3.100

Table S2. Statistical test on RT-qPCR

***MYC* (P1 expression) RT-qPCR**	**P-value**	**Welch-corrected, two-tailed, student t-test**
MYC WT/MYC MUT	<0.0001 (****)	t=155.6, df=5.000
MYC WT/MYC SWP	0.0218 (*)	t=6.658, df=2.000
***MYC* (Total expression) RT-qPCR**	**P-value**	**Welch-corrected, two-tailed, student t-test**
MYC WT/MYC MUT	0.0277 (*)	t=3.429, df=3.000
MYC WT/MYC SWP	0.7384 (ns)	t=0.3638, df=3.000
**Clones *MYC* (P1 expression) RT-qPCR**	**P-value**	**Welch-corrected, two-tailed, student t-test**
MYC WT/MYC MUT	<0.0001 (****)	t=18.97, df=3.601
MYC WT/MYC SWP	0.0138 (*)	t=3.478, df=5.592
MYC WT/MYC MIN	0.007 (***)	t=15.75, df=2.869
MYC WT/MYC FLIP	0.0899 (ns)	t=2.617, df=2.6
**Clones *MYC* (Total expression) RT-qPCR**	**P-value**	**Welch-corrected, two-tailed, student t-test**
MYC WT/MYC MUT	0.0417 (*)	t=3.885, df=2.396
MYC WT/MYC SWP	0.3449 (ns)	t=1.051, df=4.635
MYC WT/MUT MIN	0.0488 (*)	t=2.832, df=3.890
MYC WT/MYC FLIP	0.05881 (ns)	t=0.6041, df=3

Table S3. Statistical test on Counts per million (CPM) at the *MYC* G4 locus

MYC (G4) coordinates: (chr8: (-) 127,735,928-127,735,954)

**MLL1 CUT&Tag**	**P-value**	
MYC WT/MYC MUT	0.0112 (*)	t=5.142, df=3.310
MYC WT/MYC SWP	0.2336 (ns)	t=1.635, df=2.175

MLL4 CUT&Tag	P-value	
MYC WT/MYC MUT	0.0331 (*)	t=3.957, df=2.781
MYC WT/MYC SWP	0.7240 (ns)	t=0.3791, df=3.971

Table S4. sgRNA for MYC G4 genome editing

sgRNA	PAM	Sequence	Cut site
MYCG4-sgRNA	GGG	ACCCGGGAGGGGCGCTTATG	Chr8: (+) 127,735,955-127,735,955

Table S5. CRISPR repair templates for the generation of G4 genetic edits

Homology Repair Template	Sequence
HDR_MutMYC_ssDNA	CGTAGTTAATTCATGCGGCTCTCTTACTCTGTTTACATCCTAGAGCTAGAGTGCTCGGCTGCCCGGCTGAGTCTCCTCCTCACCTTGTTCACGCTCTTAACGCTCTTAATAAGCGCGGATCCTAGGTTCCCAAAGCAGAGGGCGTGGGGGAAAAGAAAAAAGATCCTCTCTCGCTAATCTCCGCCCACCGGCCCTTTATA
HDR_MutMIN_ssDNA	CGTAGTTAATTCATGCGGCTCTCTTACTCTGTTTACATCCTAGAGCTAGAGTGCTCGGCTGCCCGGCTGAGTCTCCTCCTCACCTTCTCCACGCTCCGGATCCTCTCCATAAGCGCTCCTCGCGGGTTCCCAAAGCAGAGGGCGTGGGGGAAAAGAAAAAAGATCCTCTCTCGCTAATCTCCGCCCACCGGCCCTTTATA
HDR_KRas32R_ssDNA	CGTAGTTAATTCATGCGGCTCTCTTACTCTGTTTACATCCTAGAGCTAGAGTGCTCGGCTGCCCGGCTGAGTCTCCTCCTCGCTGCCTCCCCCTCTTCCCTCTTCCCACACCGCCCTCAGCCGCGGTTCCCAAAGCAGAGGGCGTGGGGGAAAAGAAAAAAGATCCTCTCTCGCTAATCTCCGCCCACCGGCCCTTTATA
HDR_MYCFlip_ssDNA	CGTAGTTAATTCATGCGGCTCTCTTACTCTGTTTACATCCTAGAGCTAGAGTGCTCGGCTGCCCGGCTGAGTCTCCTCCGGGAGGGGCGCTTATGGGGAGGGTGGGGAGGGTGGGGAAGGTGGGGGTTCCCAAAGCAGAGGGCGTGGGGGAAAAGAAAAAAGATCCTCTCTCGCTAATCTCCGCCCACCGGCCCTTTATA

Table S6. Primers for amplicon Sanger sequencing

Oligonucleotide	Sequence (5' to 3')
MYC Forward – Genotyping	GGGAGTTTATTCATAACGCGCT
MYC Reverse – Genotyping	TCGCTGGAATTACTACAGCGAG
AGAP2-AS1/AGAP2_chr12_58122064_Forward	TCGTCGGCAGCGTCTCCACTCCACCTCAAACTCT
AGAP2-AS1/AGAP2_chr12_58122064_Reverse	GTCTCGTGGGCTCGGAAAGCAGGTAACAAGTGGGG
CAMTA1|RP11-334N17.1_chr1_6991076_Forward	TCGTCGGCAGCGTCTCTTAGCCCACTCTTCACCA
CAMTA1|RP11-334N17.1_chr1_6991076_Reverse	GTCTCGTGGGCTCGGCTTTCCTCGCCGAACACATA
CEP250_chr20_34097139_Forward	TCGTCGGCAGCGTCGAACGGAGGAAGCTGAAGAG
CEP250_chr20_34097139_Reverse	GTCTCGTGGGCTCGGTCAAGCTGTGATCAAGCTCC
ELL_chr19_18571437_Forward	TCGTCGGCAGCGTCGAGGCACTGAACCAAAGTCA
ELL_chr19_18571437_Reverse	GTCTCGTGGGCTCGGTGTGGTGGAGGAAGAGACAT
IQSEC1_chr3_12963763_Forward	TCGTCGGCAGCGTCCAGTCTTTGGTCCATCCTCG
IQSEC1_chr3_12963763_Reverse	GTCTCGTGGGCTCGGCTGGGAAAACTGCCAGCATA
NTRK3|NTRK3-AS1_chr15_88801037_Forward	TCGTCGGCAGCGTCGGATCGGGGAAGGAAGAAAC
NTRK3|NTRK3-AS1_chr15_88801037_Reverse	GTCTCGTGGGCTCGGGGACTCCCCTAACCCTTTCT
RP5-1177M21.1|LINC00237_chr20_21010128_Forward	TCGTCGGCAGCGTCTAGCCAGGCTCTATACGCTT
RP5-1177M21.1|LINC00237_chr20_21010128_Reverse	GTCTCGTGGGCTCGGGTGTGTGTGTAGTGGTGGTT
RPSAP56|AC009120.4_chr16_73980328_Forward	TCGTCGGCAGCGTCATAGGAGAGTCCCCATTGCA
RPSAP56|AC009120.4_chr16_73980328_Reverse	GTCTCGTGGGCTCGGTGAGCCTGACTTGGTAGGAA
SESN1|RNU6-653P_chr6_109331389_Forward	TCGTCGGCAGCGTCTCTGACAGCACCAGGAGTAA
SESN1|RNU6-653P_chr6_109331389_Reverse	GTCTCGTGGGCTCGGTACTGACACAGGACTGGAGG
SIGIRR|ANO9_chr11_417767_Forward	TCGTCGGCAGCGTCCTCTTCCCTTTCCTCCAGGA
SIGIRR|ANO9_chr11_417767_Reverse	GTCTCGTGGGCTCGGAAACTGGCGCAAATTTCCTG

Table S7. RT-qPCR primers

RT-qPCR Oligonucleotide	Sequence (5' to 3')
MYC Forward	CAGGACTGTATGTGGAGCGG
MYC Reverse	GTCGTTGAGAGGGTAGGGGA
GAPDH Forward	GTCTCCTCTGACTTCAACAGCG
GAPDH Reverse	ACCACCCTGTTGCTGTAGCCAA
P1 Forward	CTTGGCGGGAAAAAGAACGG
P1 Reverse	AGTTAGATAAAGCCCCGAAAACC

Table S8. Circular dichroism assay oligos

CD Oligonucleotide	Sequence (5' to 3')
MYC WT (27bp)	TGGGGAGGGTGGGGAGGGTGGGGAAGG
MYC MUT (27bp)	TTAAGAGCGTTAAGAGCGTGAACAAGG
MUT MIN (27bp)	TGGAGAGGATCCGGAGCGTGGAGAAGG
MUT CORE (27bp)	TGGAGAGGATCCGGAGCGTGGAGAAGG
KRAS SWP (32bp)	AGGGCGGTGTGGGAAGAGGGAAGAGGGGGAGG
MYC WT (48bp)	CGGGAGGGGCGCTTATGGGGAGGGTGGGGAGGGTGGGGAAGGTGGGGA
MYC MUT (48bp)	TAGGATCCGCGCTTATTAAGAGCGTTAAGAGCGTGAACAAGGTGAGGA
MUT MIN (48bp)	CGCGAGGAGCGCTTATGGAGAGGATCCGGAGCGTGGAGAAGGTGAGGA
MUT CORE (48bp)	CGGGAGGGGCGCTTATGGAGAGGATCCGGAGCGTGGAGAAGGTGGGGA
KRAS SWP (48bp)	GCGGCTGAGGGCGGTGTGGGAAGAGGGAAGAGGGGGAGGCAGCGAGGA
Permutation 1 (27 bp)	TGAGGAGGGTGGGGAGGGTGGGGAAGG
Permutation 2 (27 bp)	TGGGGAGGGTGAGGAGGGTGGGGAAGG
Permutation 3 (27 bp)	TGGGGAGGGTGGGGAGAGTGGGGAAGG
Permutation 4 (27 bp)	TGGGGAGGGTGGGGAGGGTGGGGAATT
Permutation 5 (48 bp)	CGGGAGGGGCGCTTATGAGGAGGGTGGGGAGGGTGGGGAAGGTGGGGA
Permutation 6 (48 bp)	CGGGAGGGGCGCTTATGGGGAGGGTGAGGAGGGTGGGGAAGGTGGGGA
Permutation 7 (48 bp)	CGGGAGGGGCGCTTATGGGGAGGGTGGGGAGAGTGGGGAAGGTGGGGA
Permutation 8 (48 bp)	CGGGAGGGGCGCTTATGGGGAGGGTGGGGAGGGTGGGGAATTTGGGGA
Permutation 9 (48 bp)	CGGGAGGGGCGCTTATGGGGAGGGTGGGGAGGGTGGAGAAGGTGGGGA
Permutation 10 (48 bp)	CGGGAGGGGCGCTTATGGGGAGGGTGGGGAGCGTGGGGAAGGTGGGGA
Permutation 11 (48 bp)	CGGGAGGGGCGCTTATGGGGAGGGTCCGGAGGGTGGGGAAGGTGGGGA
Permutation 12 (48 bp)	CGGGAGGGGCGCTTATGGAGAGGGTGGGGAGGGTGGGGAAGGTGGGGA
Permutation 13 (48 bp)	CGGGAGGGGCGCTTATGGGGAGGGTGCGGAGGGTGGGGAAGGTGGGGA
Permutation 14 (48 bp)	CGGGAGGGGCGCTTATGGGGAGGATCCGGAGGGTGGGGAAGGTGGGGA
Permutation 15 (48 bp)	CGGGAGGGGCGCTTATGGGGAGGATCCGGAGGGTGGAGAAGGTGGGGA
Permutation 16 (48 bp)	CGGGAGGGGCGCTTATGGGGAGGATCCGGAGCGTGGGGAAGGTGGGGA
Permutation 17 (48 bp)	CGGGAGGGGCGCTTATGGAGAGGATCCGGAGGGTGGGGAAGGTGGGGA
Permutation 18 (48 bp)	CGGGAGGGGCGCTTATGGAGAGGATCCGGAGCGTGGAGAAGGTGGGGA
Permutation 19 (48 bp)	CGCGAGGAGCGCTTATGGAGAGGATCCGGAGCGTGGAGAAGGTGAGGA
Permutation 20 (48 bp)	TAGGATCCGCGCTTATTAAGAGCGTTAAGAGCGTGAACAAGGTGAGGA

Table S9. CUT&Tag primary antibodies and Chem-map probes

Primary Antibody	RRID	Catalog
BG4	n/a	Self-made
H3K4me1	AB_306847	Abcam, ab8895
H3K4me3	AB_306649	Abcam, ab8580
H3K27me3	AB_2616029	Cell signaling, 9733

MLL1 (KMT2A)	AB_11212995	EMD Millipore, 05-765
MLL4 (KMT2D)	n/a	ABE1867
CNBP	AB_2882413	Proteintech, 67109-Ig
SP1	AB_10898171	Proteintech, 21962-1-AP
PDS-biotin	n/a	Self-made
SG4	n/a	Self-made
SG4-R105A mutant	n/a	Self-made
RNAPIIS5P	AB_2798246	Cell signaling, 13523
RNAPIIS2P	AB_2798238	Cell signaling, 13499
R-loop (S9.6)	AB_2861387	EMD Millipore, MABE1095

Table S10. CUT&Tag secondary and tertiary antibodies

Secondary/Tertiary Antibodies	RRID	Catalog
Anti-Flag	AB_2217020	Cell Signaling, 2368S
Anti-Rabbit	AB_10775589	Antibodies online, ABIN101961
Anti-Mouse	AB_2614925	Abcam, Ab46540

Table S11. CUT&Tag qPCR Primers

Primer	Sequence (5' to 3')
MYC Forward	GAGCTAGAGTGCTCGGCTG
MYC Reverse	GTGGGCGGAGATTAGCGAG
MAZ Forward	ACTCAGCGCAGGATTGTAAATA
MAZ Reverse	CCTCATGCTTCGGCTTCC
RPA3 Forward	CGGAAGTTGACAGATACAGGG
RPA3 Reverse	GATCGCAGAAAGGTAGTCTCAG
RBBP4 Forward	GAAAGCTACTCCGCGCGTCT
RBBP4 Reverse	ACCTTCGCGCCAACATCAG

Table S12. Pull-down and BLI assay oligos

PD Oligonucleotide	Sequence (5' to 3')
MYC G4	TTTTTGGGGAGGGTGGGGAGGGTGGGGAAGG
MYC MUT	TTTTTTAAGAGCGTTAAGAGCGTGAACAAGG
KRAS G4	TTTTAGGGCGGTGTGGGAAGAGGGAAGAGGGGGAGG

Table S13. KAS-qPCR primers

KAS Oligonucleotide	Sequence (5' to 3')
G4 KAS FWD	AGCTAGAGTGCTCGGCTG
G4 KAS REV	CGGAGATTAGCGAGAGAGGAT
GAPDH KAS FWD	TACTAGCGGTTTTACGGGCG
GAPDH KAS REV	AGGCTGCGGGCTCAATTTAT
NANOG KAS FWD	TCCCATTCCTGTTGAACCATATT
NANOG KAS REV	TCCCGTCTACCAGTCTCACC

Table S14. KAS-qPCR data (calculated % input and statistical analyses)

Calculated % input				
Cell line		*MYC*	*GAPDH*	*NANOG*
MYC WT		19.09056476	108.880938	1.04496362
		24.46121597	97.2707107	0.8124155
MYC MUT		3.222929904	120.978086	0.44647049
		3.576511132	113.799626	1.00768497
		5.03485809	135.177889	1.32728539
		3.022402433	75.8577751	0.70364778
		4.176839156	107.440483	0.7671225
		3.729126023	108.796671	0.97595138
KRAS SWP		19.27703249	110.122936	1.12998041
		24.56864847	75.3385119	0.66893106
		19.86322789	135.568003	0.68724134
		9.870726152	77.46145	0.41828988
		21.10126548	76.5324864	0.40656318
		23.06877483	126.41074	1.19056841
**Unpaired t-test**				
WT vs MUT	P-value	0.000023	0.479433	0.528904
	t ratio	7.423	0.7346	0.6523
	df	10.00	10.00	10.00
MUT vs SWP	P-value	0.000012	0.644014	0.812976
	t ratio	13.05	0.4863	0.2472
	df	6.000	6.000	6.000
WT vs SWP	P-value	0.615131	0.894699	0.516717
	t ratio	0.5300	0.1381	0.6887
	df	6.000	6.000	6.000

Table S15. DEseq2 analysis on RNA-seq

Comparison of gene expression (fold changes) between MYC MUT and KRAS SWP compared to control revealed by DEseq2 analysis on the RNA-seq dataset.

Gene ID	Fold Change	P-value	q-value
MYC overall expression (MYC WT vs MYC MUT)	0.49	2.76E-16	9.00E-15
MYC overall expression (MYC WT vs KRAS SWP)	0.96	0.7448	0.9692 (ns)
MYC P1 transcript (MYC WT vs MYC MUT)	0.27	4.46E-27	4.53E-27
MYC P1 transcript (MYC WT vs KRAS SWP)	0.96	0.1758	0.3516 (ns)

Additional methods

CRISPR/Cas9 design

For transfection-based editing, the guide RNA construct was generated as a separate sgRNA plasmid using pSpCas9(BB)-2A-GFP (PX458) as backbone (Addgene, cat #48138). The sgRNA sequence was synthesized using complementary ssDNA oligonucleotides (Sigma-Aldrich) with overhangs containing the BbsI restriction site (New England Biolabs, cat #R3539), which were used for cloning into the expression plasmid. Plasmid DNA constructs were validated by Sanger sequencing (Source Bioscience). For electroporation, 400,000 HEK293T cells were electroporated (Amaxa 4D Nucleofector unit, Lonza) with 4 μg TrueCut SpCas9 protein V2 (Invitrogen, cat #A36498), 80 pmol guide RNA (Synthego) and 20 pmol single-stranded HRT donor using the program CM-130 in SF nucleofector solution (Lonza). Cells were re-seeded in 6-well plates (Costar®) and sub-cloned 72 h after electroporation. The top ten CRISPOR predicted off-target sites were analysed by amplicon Sanger sequencing (table S6). Editing efficiencies were determined from Sanger sequencing profiles of bulk edited cell populations using Synthego's Inference of CRISPR Edits (ICE) program (https://ice.synthego.com).

Cell line characterization and genotyping strategy

WT and clonal populations of edited cell lines were genotyped by Sanger sequencing (Source BioScience) of an 822bp PCR amplicon spanning the edited region (chr8: 127,735,433 – 127,736,254) (fig. S4). PCR primers for genotyping are listed in table S6. Homozygous clones containing the desired edits with no unexpected point mutations, deletions or insertions in the target site and flanking regions were selected for this study.

Cell culture

For sub-culturing cells were washed with DPBS pH 7.4 (Gibco™, 14190094), detached with StemPro™ Accutase™ (Gibco™, cat #A11105-01) at 37°C for 5 min, and resuspended in 10-fold excess of growth media. J1 (strain 129S4/SvJae) (8) mouse embryonic stem cell (mESC) cultures were routinely maintained in high-glucose DMEM (Sigma-Aldrich, D6546) containing 10% FBS (Gibco™, cat #16141079), 1X GlutaMax-I (Gibco™, cat #35050-038), 1X NEAA (Gibco™, cat

#11140-035), 0.1 mM β-mercaptoethanol (Sigma-Aldrich, cat #M3148 diluted to 50 mM in 50 µM EDTA/PBS solution), 1000 U/mL recombinant LIF (PeproTech, murine LIF, cat #250-02), 1 µM PD (Sigma-Aldrich, cat #PZ0162-5MG) and 3 µM CHIR (Cambridge biosciences, cat #HY-10182A-5MG). Cells were cultured under standard conditions at 37°C in water-saturated, CO_2-enriched (5%) atmosphere on 0.2% (w/v) gelatine-coated (Sigma-Aldrich, cat #G9391) plates and regularly tested for mycoplasma contamination (RICS, CRUK Cambridge Institute). Authentication of mESCs was performed by mouse STR analysis (RICS, CRUK Cambridge Institute). For passaging, cells were washed with PBS (Gibco™, cat #14190094), detached with TrypLE (Gibco™, cat #12604-013) at 37°C for 5 min and resuspended in a 10-fold excess of mESC growth medium.

Cleavage under targets and tagmentation (CUT&Tag) and Chem-map

500,000 HEK293T cells or 200,000 mESCs were fixed with 0.1% formaldehyde in PBS for 2 mins at room temperature and quenched with glycine. Fixed cells were centrifuged at 400 x g for 3 mins and resuspended in wash buffer (20 mM HEPES pH 7.5, 150 mM KCl, 0.5 mM spermidine in nuclease-free water with a Roche Complete Protease Inhibitor EDTA-free tablet). CUT&Tag experiments were performed on Concanavalin A (ConA)-coated magnetic beads (Bang Laboratories, cat #BP531) or ConA-conjugated Dynabeads™ MyOne™ Streptavidin T1 beads (ThermoFisher Scientific, cat #65601). For MyT1 beads preparation, beads were washed twice with PBS pH 6.8, resuspended in 1xPBS pH 6.8 supplemented with 0.01% Tween-20 and mixed with biotin-conjugated ConA (Sigma-Aldrich, cat #C5275-5MG) solution at 2.3 mg/mL at a 1:0.5 ratio. The mix was then incubated at 22°C at 400 rpm for 30 min.

SG4 and SG4mut (R105A) CUT&Tag were performed in extracted nuclei as previously described (9). Aliquots of 500,000 cells/100 µL were added to 10 µL of beads, washed twice with 100 µL wash buffer and resuspended in 1% BSA Antibody Buffer. As R-loop CUT&Tag controls, RNase A and RNase H treatments were performed prior to primary antibody incubation. 10 µg of RNase A (Takara) or 100 U RNase H (New England Biolabs, cat #M0297S) in 50 µL Dig-wash buffer were added to the cells and incubated at 37°C for 40 min.

CUT&Tag and Chem-map-qPCR

Primers for *MYC* were designed using NCBI Primer-BLAST, with parameters (i) PCR product size between 70 and 200 bp, (ii) Primer melting temperature: minimum of 57 °C, optimal of 60°C and maximum of 63°C and (iii) exon junction match (min 5' match of 7 bp, min 3' match of 4 bp and max 3' match of 8 bp). Primer suitability for qPCR was initially validated by ensuring that primer locations were not in Tn5 tagmentation sites based on generated paired-end sequencing data. For Chem-map and CUT&Tag-qPCR quantification.

Sequencing data processing

Illumina sequencing paired end output files were demultiplexed using demux Illumina version 3.0.9 using the flags; -c -d -i -e -t 1 -r 0.01 -R -l 9. The resulting fq.gz files underwent sequencing quality control using FastQC v0.11.8, and their summary was visualized by MultiQC v1.11. Bases with a quality score below 20 were trimmed from both reads using cutadapt (cutadapt -q 20). Fastq files were aligned to the combined hg38 and *E. coli* genomes (or the corresponding genome builds which include the modified site confirmed by Sanger sequencing) using bwa 0.7.17-r1188 with only reads in the whitelist regions of hg38 continuing the process pipeline. Duplicates were removed using Picard version 2.20.3 (Picard MarkDuplicates). Peaks were called using SEACR version 1.3, without input control, reporting the top 1% by area under curve (AUC) regions, using both the relaxed and stringent criteria. Assessment of peak overlap across biological replicates was performed using intervene upset and pairwise modules. High reproducibility across samples was confirmed (fig. S22 and S23). BigWig files were created using deepTools version 2.0 bamCoverage using the mapped BAM files and the flags --binSize 3 --normalizeUsing CPM --extendReads, for fragments of up to 350 or 600 nt (using –maxFragmentLength). Additionally, RNAPolII CUT&Tag libraries were rescaled for spike in normalization to 1,000,000/(number of *E. coli* reads).

RNA-seq data processing

Human genome reference hg38 fasta file was downloaded from UCSC database. Genomic annotations (gtf file) were downloaded from Gencode project portal (https://ftp.ebi.ac.uk/pub/databases/gencode/Gencode_human/release_42). The overall quality of sequencing reads was evaluated using FastQC (v.0.11.9). Sequence alignments to the reference human genome (GRCh38) were performed using bwa mem (bwa version:0.7.17) with default parameters. Sorted bam files were generated with SAMtools (version 1.15.1). Deduplication of the resulting mapped reads was performed with Picard MarkDuplicates (v2.18.7) (http://broadinstitute.github.io/picard/). Differential gene expression analysis was performed with the DESeq2 package in R (version 1.38.0) on the raw read counts (table S15). Genes with an average of fewer than 10 reads per sample were omitted from downstream analysis. Benjamini & Hochberg correction was performed with significance thresholds $|\log2FoldChange| > 0.5$ and adjusted P-value $(Padj) < 0.05$. Protein–protein interaction (PPI) network for the differentially expressed genes (DEGs) was constructed using the STRING plugin in Cytoscape using the parameters, degree cut-off = 2, node score cut-off = 0.2, k-score = 2, and Max Depth = 100. Hub genes were determined in the densest connected region using the degree algorithm in CytoHubba. A darker colour means a higher degree score. Pathway enrichment was performed using Gene set enrichment analysis (GSEA) (https://www.gsea-msigdb.org/gsea/index.jsp) with pathway definitions from two gene set collections, namely REACTOME, and the hallmark gene set.

Supplementary references

1. A. T. Phan, Y. S. Modi, D. J. Patel, Propeller-type parallel-stranded G-quadruplexes in the human c-myc promoter. *J. Am. Chem. Soc.* **126**, 8710-8716 (2004).
2. A. M. Fleming, J. Zhou, S. S. Wallace, C. J. Burrows, A Role for the Fifth G-Track in G-Quadruplex Forming Oncogene Promoter Sequences during Oxidative Stress: Do These "Spare Tires" Have an Evolved Function? *ACS Cent. Sci.* **1**, 226-233 (2015).
3. H. You, J. Wu, F. Shao, J. Yan, Stability and Kinetics of c-MYC Promoter G-Quadruplexes Studied by Single-Molecule Manipulation. *J. Am. Chem. Soc.* **137**, 2424-2427 (2015).
4. J. Kypr, I. Kejnovská, D. Renčiuk, M. Vorlíčková, Circular dichroism and conformational polymorphism of DNA. *Nucleic Acids Res.* **37**, 1713-1725 (2009).
5. A. I. Karsisiotis *et al.*, Topological Characterization of Nucleic Acid G-Quadruplexes by UV Absorption and Circular Dichroism. *Angew. Chem., Int. Ed.* **50**, 10645-10648 (2011).
6. E. Hatzakis, K. Okamoto, D. Yang, Thermodynamic stability and folding kinetics of the major G-quadruplex and its loop isomers formed in the nuclease hypersensitive element in the human c-Myc promoter: effect of loops and flanking segments on the stability of parallel-stranded intramolecular G-quadruplexes. *Biochemistry* **49**, 9152-9160 (2010).
7. J.-L. Mergny, L. Lacroix, UV Melting of G-Quadruplexes. *Curr. Protoc. Nucleic Acid Chem.* **37**, 17.11.11-17.11.15 (2009).
8. E. Li, T. H. Bestor, R. Jaenisch, Targeted mutation of the DNA methyltransferase gene results in embryonic lethality. *Cell* **69**, 915-926 (1992).
9. S. Galli *et al.*, DNA G-Quadruplex Recognition In Vitro and in Live Cells by a Structure-Specific Nanobody. *J. Am. Chem. Soc.* **144**, 23096-23103 (2022).
